# Evolution of loss of heterozygosity patterns in hybrid genomes of *Candida* yeast pathogens

**DOI:** 10.1186/s12915-023-01608-z

**Published:** 2023-05-11

**Authors:** Verónica Mixão, Juan Carlos Nunez-Rodriguez, Valentina del Olmo, Ewa Ksiezopolska, Ester Saus, Teun Boekhout, Attila Gacser, Toni Gabaldón

**Affiliations:** 1 Life Sciences Department, Barcelona Supercomputing Center (BSC), Jordi Girona, 29, 08034 Barcelona, Spain; 2 Mechanisms of Disease Program, Institute for Research in Biomedicine (IRB), The Barcelona Institute of Science and Technology, Barcelona, Spain; 3 Present address: Genomics and Bioinformatics Unit, Infectious Diseases Department, National Institute of Health Dr. Ricardo Jorge, Av. Padre Cruz, 1649-016 Lisbon, Portugal; 4 Westerdijk Fungal Biodiversity Institute, Utrecht, The Netherlands; 5 Institute of Biodiversity and Ecosystem Dynamics (IBED), University of Amsterdam, Amsterdam, The Netherlands; 6 Department of Microbiology, University of Szeged, Szeged, Hungary; 7 MTA-SZTE “Lendület” Mycobiome Research Group, University of Szeged, Szeged, Hungary; 8 ICREA, Pg. Lluis Companys 23, 08010 Barcelona, Spain; 9 Centro de Investigación Biomédica En Red de Enfermedades Infecciosas, Barcelona, Spain

**Keywords:** *Candida orthopsilosis*, *Candida metapsilosis*, Hybrids, Comparative genomics, Loss of heterozygosity

## Abstract

**Background:**

Hybrids are chimeric organisms with highly plastic heterozygous genomes that may confer unique traits enabling the adaptation to new environments. However, most evolutionary theory frameworks predict that the high levels of genetic heterozygosity present in hybrids from divergent parents are likely to result in numerous deleterious epistatic interactions. Under this scenario, selection is expected to favor recombination events resulting in loss of heterozygosity (LOH) affecting genes involved in such negative interactions. Nevertheless, it is so far unknown whether this phenomenon actually drives genomic evolution in natural populations of hybrids. To determine the balance between selection and drift in the evolution of LOH patterns in natural yeast hybrids, we analyzed the genomic sequences from fifty-five hybrid strains of the pathogenic yeasts *Candida orthopsilosis* and *Candida metapsilosis*, which derived from at least six distinct natural hybridization events.

**Results:**

We found that, although LOH patterns in independent hybrid clades share some level of convergence that would not be expected from random occurrence, there is an apparent lack of strong functional selection. Moreover, while mitosis is associated with a limited number of inter-homeologous chromosome recombinations in these genomes, induced DNA breaks seem to increase the LOH rate. We also found that LOH does not accumulate linearly with time in these hybrids. Furthermore, some *C. orthopsilosis* hybrids present LOH patterns compatible with footprints of meiotic recombination. These meiotic-like patterns are at odds with a lack of evidence of sexual recombination and with our inability to experimentally induce sporulation in these hybrids.

**Conclusions:**

Our results suggest that genetic drift is the prevailing force shaping LOH patterns in these hybrid genomes. Moreover, the observed LOH patterns suggest that these are likely not the result of continuous accumulation of sporadic events—as expected by mitotic repair of rare chromosomal breaks—but rather of acute episodes involving many LOH events in a short period of time.

**Supplementary Information:**

The online version contains supplementary material available at 10.1186/s12915-023-01608-z.

## Background

The cross between two diverged lineages results in hybrid progeny carrying a chimeric genome. As a result, genomes from hybrid organisms are highly heterozygous, with a genetic divergence between pairs of homeologous chromosomes initially equal to the divergence of the two parental species [1, 2]. As suggested by Bateson [3], Dobzhansky [4], and Muller [5], this coexistence of the two diverged genomes and their respective transcripts and proteins can originate incompatibilities that negatively impact cell function and compromise the hybrid’s fitness. Despite this fitness cost, if hybridization is associated with the acquisition of a trait that provides a key advantage in a given niche, hybrid lineages may survive [6]. Under these circumstances, natural selection is expected to favor loss of heterozygosity (LOH) in genomic regions comprising heterozygous genes involved in genomic incompatibilities [1, 2]. Several processes can shape hybrid genomes, including the duplication or loss of chromosomes leading to chromosomal aneuploidies, gene loss, gene conversion, or whole-genome duplication [7–11]. These mechanisms contribute to progressive LOH and promote genome stabilization by reducing the amount of heterozygosity and genomic incompatibilities.

**Fig. 1:**
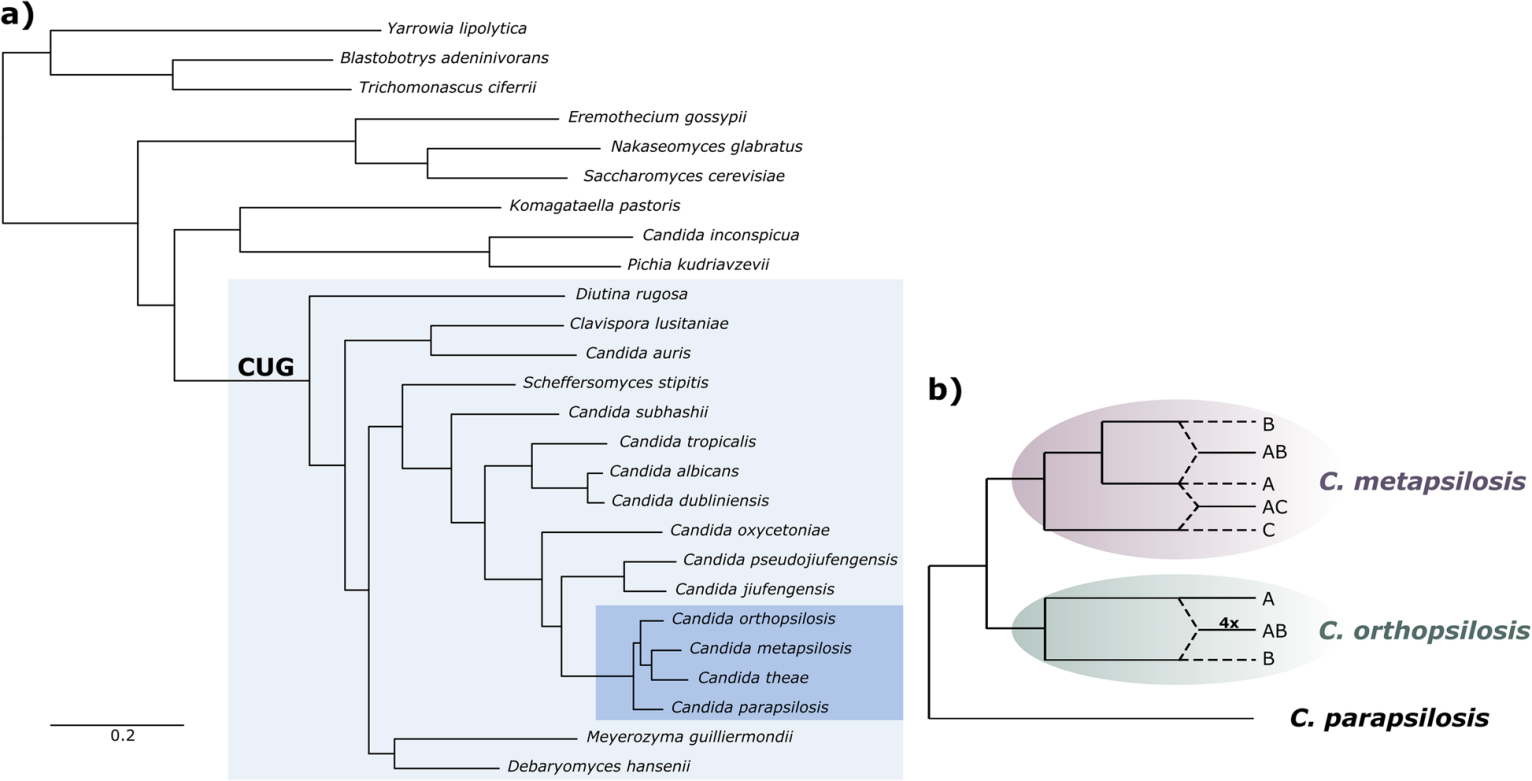
Phylogenetic position of *C. parapsilosis* species complex. **a** Maximum likelihood tree reconstruction of the concatenated amino-acid multiple sequence alignment of the phylome 866 [22] available at PhylomeDB database (http://phylomedb.org/phylome_866) [24]. The light blue background highlights the species of the CUG clade. The dark blue background highlights the *C. parapsilosis* species complex. **b** Schematic representation of the evolutionary trajectory of the three opportunistic pathogens of the *C. parapsilosis* species complex, where A, B, and C in the pink background denote the different *C. metapsilosis* parental lineages and A and B in the green background denote the two *C. orthopsilosis* parental lineages

Hybridization may occasionally lead to the emergence of new lineages or species, with some studies pointing to a role of hybridization in the emergence of new human pathogens [12–15]. Indeed, recent genomic analyses have uncovered numerous hybrids among opportunistic fungal human pathogens [12–22]. For instance, *Candida parapsilosis* species complex comprises three opportunistic pathogenic species, from which two, *Candida orthopsilosis* and *Candida metapsilosis*, have hybrid strains (Fig. 1) [12, 16, 18, 21, 23]. All *C. metapsilosis* strains analyzed thus far are hybrids, and all but one are inferred to descend from the same hybridization event between two unknown lineages (A and B) with 4.5% divergence at the nucleotide level (clade 1) [12, 21]. The remaining strain (MSK414), although classified as *C. metapsilosis*, was recently shown to descend from an independent hybridization event involving one of the parentals of the previously mentioned clade and an alternative parent C with 4.7% divergence at the nucleotide level (clade 2) [21]. In *C. orthopsilosis*, most analyzed strains are hybrids, and the few known homozygous strains always correspond to the same parental lineage [16, 18], being the alternative parental currently unknown. In this case, at least four independent hybridization events have been inferred, all involving the same two lineages with 4.5% nucleotide divergence [16, 18]. This indicates a high propensity of this species complex to form hybrids that can successfully colonize and cause disease in humans [22]. The fact that homozygous strains from the parental lineages are relatively less frequent in the clinical setting, as compared to the hybrids, prompted the hypothesis that hybridization events between non-pathogenic or mildly pathogenic species led to the emergence of new hybrid lineages with increased virulence towards humans [1, 12].

Given the importance of hybrids, not only for the clinics but also for industry and biotechnology [1, 25, 26], multiple studies have sought to understand the evolution of hybrid genomes, with a special focus on the possible sources of genomic incompatibilities and on the mechanisms of genome stabilization [27–31]. In this respect, suboptimal interactions between nuclear and mitochondrial components, or among ribosomal subunits when proteins are encoded by different parental genomes, have been proposed as important sources of genomic incompatibilities that compromise hybrids’ fitness [27, 28, 32]. The availability of dozens of fully sequenced genomes of strains from independently formed hybrids in the *C. parapsilosis* species complex provides a unique opportunity to study the genomic aftermath of hybridization and test some of the abovementioned theoretical predictions. For instance, if the resolution of genomic incompatibilities is indeed a strong selective force, one would expect that LOH blocks among naturally occurring isolates are not randomly distributed along the genome, but rather enriched in regions comprising certain genes, such as those encoding ribosomal or mitochondrial proteins [27, 28]. To test this hypothesis and to gain insight into the evolutionary aftermath of hybridization at the genomic level, we undertook a comparative analysis of 43 publicly available, and 17 newly sequenced strains from the *C. parapsilosis* species complex.

## Results

### Genetic and geographical structure of hybrid clades and analysis of the first environmental *C. metapsilosis* hybrid

To assess genome evolution following hybridization, we analyzed publicly available genomic data for *C. orthopsilosis* and *C. metapsilosis* [12, 16, 18, 21, 33], and sequenced additional strains from these species (see the “Methods” section). We produced an improved assembly for *C. metapsilosis* based on a hybrid approach combining long- and short-read sequencing technologies (see the “Methods” section) and compared available reference genomes for both species to choose the best reference [12, 34–36] (Additional file 1). Of note, the *C. metapsilosis* genome assembly generated by this work comprises 9 scaffolds, being close to chromosome level, and represents a significant improvement over the previously available ones (Additional file 1) [12, 36]. In addition, we used re-sequencing data from the same strain obtained from two different laboratories as technical replicates to validate and improve the methodology to define LOH blocks, i.e., continuous sequences in the genome presenting a low density of heterozygous SNPs (see the “Methods” section, Additional file 2).

**Fig. 2:**
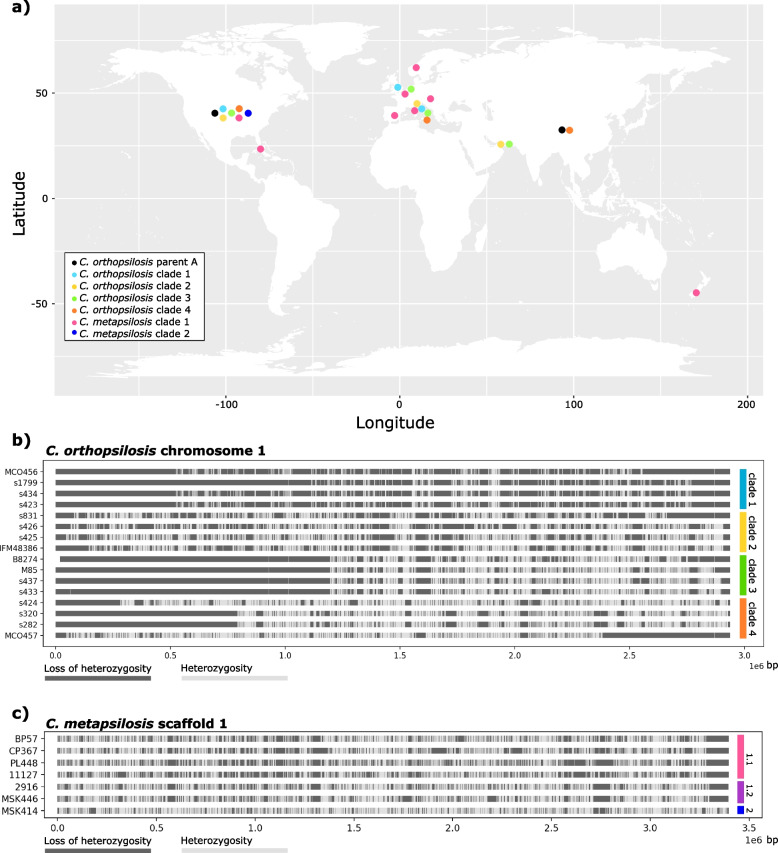
*C. orthopsilosis* and *C. metapsilosis* isolates. **a** Geographical distribution of *C. orthopsilosis* and *C. metapsilosis* isolates. Circles, with colors representing different clades, are placed on the country where at least one sample of a given clade was isolated (Additional file 24: Table S1). **b** Distribution of loss of heterozygosity (LOH, dark gray) and heterozygosity (light gray) regions in chromosome 1 of four randomly selected strains of each *C. orthopsilosis* hybrid strain. *C. orthopsilosis* clade 1 is marked in blue, clade 2 in yellow, clade 3 in green, and clade 4 in orange. **c** Distribution of loss of heterozygosity (LOH, dark gray) and heterozygosity (light gray) regions in scaffold 1 of four randomly selected strains of *C. metapsilosis* clade 1.1, the two isolates of clade 1.2, and the only strain isolated thus far of clade 2. *C. metapsilosis* clade 1.1 is marked in pink, clade 1.2 in purple, and clade 2 in dark blue

The final dataset comprised 41 *C. orthopsilosis* and 19 *C. metapsilosis* strains with a broad geographical distribution (Fig. 2a), and most notably included the first sequenced *C. metapsilosis* strain isolated from an environmental source (Additional file 24: Table S1, and Additional files 3 and 4). Our results show that all *C. metapsilosis* and the majority (36 out of 41) of *C. orthopsilosis* sequenced isolates are hybrids, consistent with previous studies [12, 18, 21]. The similarity in LOH patterns, i.e., the set of LOH blocks present in a specific genome, can reveal whether two strains likely result from the same hybridization event (Fig. 2b, c) [12]. Overall, for the shared strains, pairwise comparisons of LOH patterns agreed with the *C. orthopsilosis* clades previously described [18], except for clade 4 where the high level of heterozygosity and scarcity of LOH blocks challenge this type of comparison (Fig. 2b and Additional file 21: Fig. S1). Based on this analysis, we assigned the newly sequenced hybrid strains into existing clades (Additional file 24: Table S1).

**Fig. 3:**
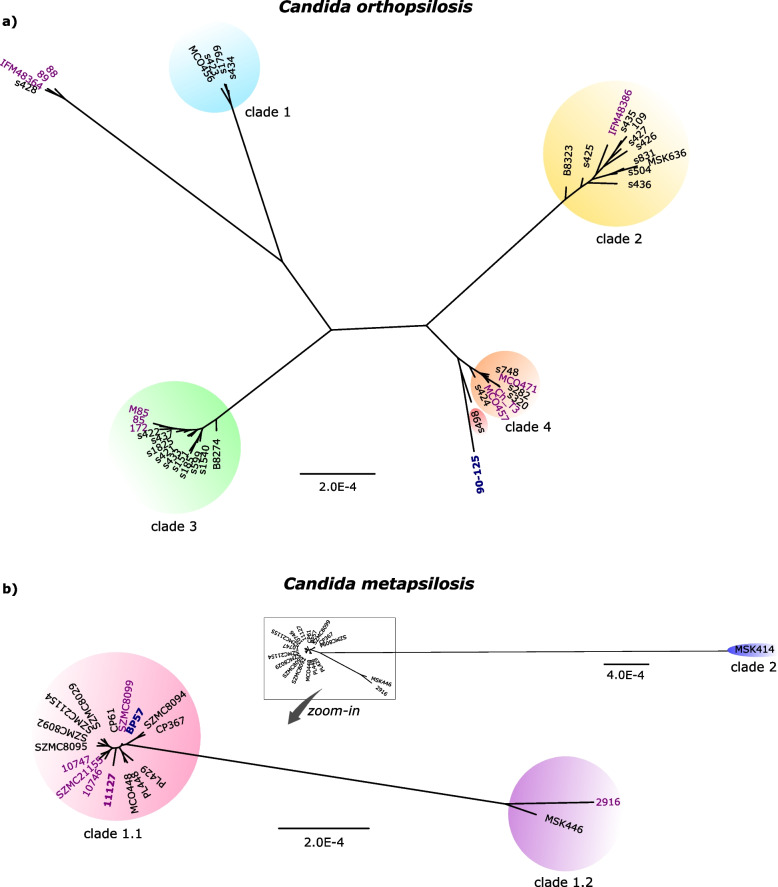
Maximum likelihood phylogenetic trees. **a ***C. orthopsilosis* tree with clade 1 highlighted with a blue background, clade 2 with yellow, clade 3 with green, and clade 4 with orange. Due to its dubious clade adscription, s498 has a red background. Isolates with no background correspond to parent A. **b** On the top right, *C. metapsilosis* main tree with clade 1 indicated with a square and clade 2 indicated with blue background. An additional zoom-in tree with strains of clade 1.1 with pink background and strains of clade 1.2 with purple background is presented. Newly sequenced strains are written in purple, and the environmental isolate is also in bold. Both reference strains are in blue

We next inferred a molecular phylogeny for *C. orthopsilosis* strains from genome-wide polymorphisms (Fig. 3a, see the “Methods” section). The phylogeny broadly supported previously defined clades, and the above-suggested clade adscription, and revealed that all new homozygous strains of *C. orthopsilosis* were close to s428, and therefore corresponded to the same, previously known, parental lineage (parent A) [18]. Notably, all clades have a broad geographical distribution, comprising isolation sites from at least two different continents (Fig. 2a). This broad geographical distribution is also apparent in the most heterozygous (and likely more recently formed [18]) clade 4, which with our increased sampling now comprises isolates from the USA, Europe, and China.

Importantly, our sampling includes the first genome from an environmental isolate of *C. metapsilosis* (strain CBS11127). Our molecular phylogenetic analysis places this hybrid among clinical isolates of clade 1 (Fig. 3b). Other *C. metapsilosis* strains have been reported from the environment, including samples from rice plants and, consistent with the source of our isolate, marine locations [37–39]. However, our analysis of deposited ITS data from these other environmental samples revealed that they likely represent different, unidentified species from the *Candida/Lodderomyces* clade (see Additional file 22: Fig. S2).

Previous analyses of *C. metapsilosis* strains have shown the existence of two independent hybrid clades with distinct parental lineages [12, 21, 33]. The molecular phylogeny of clade 1 shows that it can also be subdivided into two groups of strains (Fig. 3b), with one subclade comprising most strains (clade 1.1), with the exception of CBS2916 and MSK446, which together form a distantly related clade (clade 1.2). The divergence between these two groups is comparable to the one observed for the different hybridization events in *C. orthopsilosis* (Fig. 3a, b). As such, we hypothesized that they could represent different hybridization events between the same two parental lineages. To assess the possible relation between these two subclades of *C. metapsilosis* clade 1, we inspected their respective *MAT* loci. Previous analyses had reported a LOH event in strains of clade 1.1 resulting in a partially overwritten *MTLa* idiomorph with *MTLα* [12]. We found that this event is also present in the strains of clade 1.2, albeit with slightly different boundaries (Additional file 23: Fig. S3a).

We next assessed the number of shared LOH blocks, with the exact same boundaries, in all *C. metapsilosis* strains of clade 1. As we aimed to determine whether the two clades had a common ancestor, we decided to look at blocks with more than 1 kb, as they are confidently assigned as LOH. We found two LOH blocks with an approximate length of 1 kb, which are shared by all *C. metapsilosis* strains of this clade, including CBS2916 and MSK446 (Additional file 23: Fig. S3b and S3c). For comparison, distinct *C. orthopsilosis* clades do not share any block longer than 1 kb with the exact same boundaries (Additional file 5). The shared LOH blocks partially overlap *MTC5* and *BPH1* genes. These genes are highly heterozygous in some *C. orthopsilosis* strains, indicating that their sequences are not particularly conserved in the *C. parapsilosis* clade. Thus, a scenario involving two hybridization events in *C. metapsilosis* clade 1 would imply either the occurrence of two convergent LOH events (plus a very similar one in the *MAT* locus) or the exchange of these genetic regions. We consider that, with the current data at hand, a shared hybridization event followed by early separation of the two subclades is the most parsimonious scenario, as it does not imply any convergent LOH. This scenario would involve a very early LOH event in the *MAT* locus followed by the separation of the two subclades, and subsequent accumulation of independent LOH events, including the extension of LOH in the *MAT* locus of clade 1.2. Nevertheless, more studies are necessary to clarify this scenario.

### Lack of strong functional trends in LOH patterns

As mentioned before, evolutionary theory frameworks predict that hybrid genomes may have genetic incompatibilities between haplotypes that can cost the hybrid’s survival. Therefore, as LOH blocks remove such negative interactions, they are expected to be selected around important genes affected by such incompatibilities. As shown by the high number of hybrid isolates, the lineages of the *C. parapsilosis* species complex seem prone to hybridize and survive after hybridization. This made us wonder about how strong are the genetic incompatibilities between these lineages and, consequently, the LOH selection. To assess the strength of selection in shaping LOH patterns, we used the inferred LOH blocks to define the level of homozygosity per gene and strain, as the fraction of that gene covered by LOH blocks (see the “Methods” section). For all clades, there was clearly a fraction of genes with high levels of LOH (Additional file 6). We tested whether the fraction of shared homozygous (100% LOH) or heterozygous (0% LOH) genes between pairs of strains from different clades was different from random expectation and found that, in most cases, they are more congruent than expected by chance (*p* < 0.01, see Additional file 7). This indicates that LOH patterns in independent clades share some level of convergence that would not be expected from random occurrence and thus provide evidence for the existence of some constraints. However, considering a minimum LOH block size of 100 bp, there was not a single gene completely within a homozygous or heterozygous block in all the studied strains. When increasing this threshold to 1 kb, five genes were in heterozygous regions in all the studied strains, namely *TFA2* (required for promoter clearance by RNA polymerase), *NTF2* (essential in nucleocytoplasmic transport), *AIM21* (involved in mitochondrial migration along actin filaments), *QCR7* (component of the ubiquinol-cytochrome c reductase complex), and *DIC1* (mitochondrial carrier family). The first three and the last two genes form, respectively, two blocks of contiguous genes. We evaluated the behavior of these genes in hybrid strains of the distantly related *C. inconspicua* [13] and found that *NTF2* and *QCR7* were in heterozygous regions in all hybrid isolates from this species. Although our results are inconclusive, this striking coincidence suggests that the heterozygosity of these genes may be constrained in *Candida* spp. hybrids.

We next tested whether LOH blocks were preferentially covering genes performing certain functions. To do so, we functionally annotated the genes and performed enrichment analyses for each independent hybridization event (see the “Methods” section). To discard the possibility that highly conserved genes could be influencing our results, we limited our analysis to genes with at least one non-synonymous variant in at least one strain. While for *C. orthopsilosis* we did not find any particular enrichment, for *C. metapsilosis*, we found that genes with more than 50% overlap with LOH blocks were enriched in cell wall- and cell surface-related functions, but only when using a LOH block length threshold of 100 bp (*p*-value < 4.46e − 04, Additional file 6).

We also tested the existence of strong mito-nuclear incompatibilities. In such a scenario, we would expect the direction of LOH (i.e., which sub-genome is retained) in mtDNA interacting proteins to follow the same direction of mitochondrial inheritance (i.e., the same parental genome would be retained to minimize negative epistatic interactions). Earlier studies have linked nuclear-encoded mitochondrial proteins (e.g., pentatricopeptide repeat proteins (PPR)) to incompatibilities in hybrid yeast species [28]. Taking advantage of the unique opportunity of having different hybrid clades originating from the same parental lineages but retaining the mitochondria from different parents (*C. orthopsilosis* clades 1 and 3 inherited the mitochondrial genome of parent A, clade 2 from parent B, and clade 4 had a recombinant mitochondrion) [18], we analyzed in detail thirteen genes predicted to encode PPR proteins in *C. metapsilosis* and *C. orthopsilosis* (Additional file 8). Our results suggest that homozygosity in some of these genes may be important, but that the specific retained allele is not so relevant for hybrid survival (details in Additional files 8 and 9). This is in agreement with what was previously suggested for the nuclear genome of these species that shows no preferential retention of any of the two parental sub-genomes [12, 16, 18], and with a recent analysis of *Saccharomyces* hybrids that revealed that the overall direction of LOH is not correlated with the direction of mitochondrial inheritance [40]. Additional results regarding PPR proteins are shown in Additional file 9. Altogether, these results show that, although the LOH patterns are not random in these hybrids, there is an apparent lack of strong selection towards specific functional categories, suggesting an important role of drift in shaping heterozygosity patterns.

### *C. orthopsilosis* LOH patterns cannot be exclusively explained by mitotic recombination

A key aspect to understand the evolution of LOH patterns is to discern the molecular mechanisms by which they originated and the rate at which they accumulate. As detailed in Table 1, three main mechanisms may underlie the occurrence of LOH, namely, mitotic recombination, DNA break-induced repair, and meiotic recombination. Each of them is expected to result in specific genomic patterns that can be used to study and infer LOH evolution (Table 1). Due to the absence of a known sexual cycle in *C. parapsilosis* species complex [16, 41], the two main mechanisms assumed to be associated with the occurrence of LOH blocks in these species are mitotic recombination and DNA break-induced repair.

**Table 1:** Genomic patterns expected for scenarios of mitotic recombination, stress-induced DNA breaks, and meiotic recombination and observed in the studied *Candida* hybrids, in terms of number of LOH events, LOH size, LOH accumulation with time, LOH correlation with chromosome size, and distribution in the chromosomes

	**Mitotic recombination**	**Stress-induced DNA breaks**	**Meiotic recombination**	**Observation in *Candida* hybrids**
Large number of blocks	No	Yes	Yes	Yes
Enrichment in large blocks	Yes	Yes	No	No
Linear accumulation with time	Yes	No	No	No
Negative correlation between LOH block and chromosome size	No	No	Yes	Sometimes
Enrichment in the telomers	No	Yes	Yes	Yes

To get a better understanding of the mechanisms of LOH acquisition in these hybrids, we set up to experimentally test the occurrence of LOH in *C. orthopsilosis* (strains 172, s424 and IFM48386) and *C. metapsilosis* (strain BP57) during mitotic recombination and DNA break-induced repair. Briefly, for each strain, a time 0 isolate was divided into four clones, of which three were subjected to 30 passages in rich medium (~ 400 generations, assuming 95 min generation time [42]), and in a parallel experiment, another clone was subjected to DNA damage using UV light, thus inducing DNA break repair (see the “Methods” section for more details). A comparative genomics analysis between these samples and the time 0 strain allowed the identification of the patterns generated by spontaneous mitotic recombination and DNA break-induced repair in these hybrids. Regarding the scenario in which we expected spontaneous mitotic recombination, only two sporadic events were detected: an extra copy of chromosome 8 in one of the triplicates of the strain s424, and a > 4-kb non-crossover event leading to LOH in one of the triplicates of BP57 (Additional file 10). This suggests the occurrence of approximately 1 LOH event every 2400 mitotic cycles (2 events in 12 lineages of ~ 400 generations each, which is equivalent to ~ 4.2 10^−4^ events per mitotic cycle), thus corresponding to a rather low spontaneous LOH rate in *Candida* hybrids. This is also supported by the absence of differences in LOH blocks between the two isolates of strain s424 sequenced in this study, and years ago by another lab (see the “Methods” section and Additional file 11).

The UV light experiment resulted in two different types of cells based on their cell size (normal colony size vs. small colonies) in all strains. Therefore, we decided to select three colonies of each sample for the genomic analysis, two corresponding to a normal colony and one to a small colony. When comparing to the respective time 0, we could identify new LOH blocks in seven out of 12 (3 colonies of each of the four strains) isolates, which could be divided into non-crossovers (i.e., LOH is flanked by the original sequences) and crossovers (i.e., LOH was extended until the end of the chromosome). Regarding the normal-size colonies, we could detect three non-crossover events in one of the isolates of strain 172 (> 600 bp, > 10 kb, and > 35 kb), one non-crossover event (~ 300 bp) accompanied by a 150-bp deletion in one isolate of strain s424, and a crossover event in one of the isolates of IFM48386 (Additional file 10). Interestingly, none of the isolates of the small colonies had a non-crossover, but all of them had at least one crossover event (Additional file 10). Furthermore, all but one crossover occurred in small colonies. Unfortunately, we could not make an association between the occurrence of crossover events and the cell size alteration, because these crossover events affected different chromosomes (and consequently different genes) in the different isolates (Additional file 10). These results reveal that, although DNA breaks are associated with a limited number of inter-homeologous chromosomal recombination, they did increase the rate of LOH formation when compared to mitotic recombination. Of note, the evolved hybrid strains that underwent LOH showed similar fitness as their respective parent strains (Additional file 12).

**Fig. 4:**
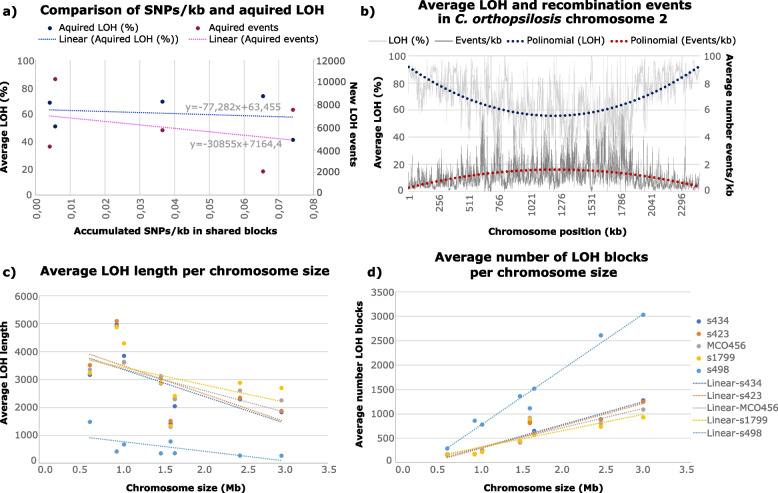
Analysis of the LOH patterns in *C. orthopsilosis* supports the occurrence of meiotic recombination. **a** Non-linear accumulation of LOH blocks with time (SNPs/kb). **b** The average percentage of LOH and number of recombination events in 1-kb windows of *C. orthopsilosis* chromosome 2, only considering the 16 randomly selected strains represented in Fig. 2b to avoid bias of different clade sampling size. **c** Negative correlation between the average LOH length and the chromosome size in *C. orthopsilosis* clade 1 and s498. **d** Positive correlation between the number of LOH blocks and the chromosome size in *C. orthopsilosis* clade 1 and s498

LOH blocks resulting from mitotic recombination and from DNA break-induced repair are expected to accumulate linearly with time. Therefore, the relative level of LOH in hybrid strains is often taken as a proxy for the relative age of different hybridization events [18]. However, it is so far unknown what is the rate at which LOH blocks accumulate, and whether they accumulate linearly with time. Each natural *C. orthopsilosis* hybrid strain experienced at least 4124 recombination events when considering LOH blocks longer than 100 bp. This number is expected to be even higher, if considering reciprocal recombination events (i.e., recombination of haplotypes without LOH), for which we found evidence in all strains of clades 1 and 2 (except s427, s436, and s504) (Additional file 13, see the “Methods’ section). Considering our observations on the evolution of *C. orthopsilosis* strains in the laboratory, and the observed similar patterns between diverged strains descending from the same hybridization event (Fig. 2b), we hypothesized that other mechanisms might be involved in the occurrence of LOH blocks in these hybrids. We tested this idea by comparing the level of accumulated LOH (in terms of percentage of the genome size and absolute number of events) with the number of heterozygous mutations within shared LOH blocks in a given clade, as both mutational events must have postdated the divergence of the clade, and they should have occurred in the same window of time for each clade (see the “Methods” section). *C. orthopsilosis* strain s498 was excluded from the analysis due to its dubious clade-adscription (Fig. 3a). In this analysis, we estimated that each *C. metapsilosis* strain of clade 1.1 accumulated on average 0.01 heterozygous SNPs per kilo-base, and *C. orthopsilosis* clades 1, 2, 3, and 4 accumulated 0.07, 0.04, 0.004, and 0.07 heterozygous SNPs per kilo-base, respectively (Additional file 14). Importantly, we did not observe any significant correlation between the estimated mutational load and the amount of LOH acquired after their divergence (Spearman rho for the LOH percentage =  − 0.1639, *p* = 0.65094; Spearman rho for the number of new LOH events =  − 0.1539, *p* = 0.80483; Fig. 4a), even when removing *C. metapsilosis* from the analysis (Spearman rho for the LOH percentage =  − 0.32602,* p* = 0.43064; Spearman rho for the number of new LOH events = 0.10541, *p* = 0.89459). These results indicate that LOH and SNPs do not accumulate proportionally, and suggest that LOH does not accumulate with time in a linear way (Fig. 4a), as it would be expected in a scenario of mitotic recombination. Therefore, the patterns found in naturally evolved *C. orthopsilosis* hybrids are at odds with the LOH patterns found in the experimentally evolved strains, and we consider that they can hardly be explained by the observed low mitotic recombination rates. We propose that other factors besides time are influencing the occurrence of LOH in hybrids of *C. parapsilosis* species complex.

### *C. orthopsilosis* hybrids have genomic patterns compatible with meiotic recombination

Considering our findings of an apparent non-linear accumulation of LOH, and taking advantage of the larger number of hybrid strains comprising our dataset, we decided to revisit a previously proposed hypothesis that meiotic recombination can occur in these lineages. Indeed, although inter-hom(e)ologous chromosome recombination can occur during mitosis [43], in this process, most chromosomal breaks are solved with sister chromatids [44]. In contrast, during meiosis, inter-hom(e)ologous chromosome recombination is an essential event, and therefore, it is expected to occur at a higher rate [45, 46]. Initial comparative genomics analyses of the first reported hybrid strain in *C. orthopsilosis* suggested that meiotic, in addition to mitotic, recombination could be in part responsible for the observed LOH patterns [16]. At that time, only two hybrid strains (MCO456 and AY2) were available. However, although the presence of alternative mating types in these hybrids suggests they originated through sexual crosses, as mentioned before, meiosis has so far not been observed in the *C. parapsilosis* species complex [16, 41].

Previous studies suggested that meiotic recombination does not homogeneously occur across the genome but rather presents higher rates in the extremities of the chromosomes [47, 48]. Patterns of LOH distribution across *C. orthopsilosis* genome revealed that these hybrids tend to have an increased average level of LOH at the edges of the chromosomes, which is accompanied by a decreased average number of recombination events in the same regions (Fig. 4b and Additional file 15). A recent study has shown that pre-existing LOH blocks in *S. cerevisiae* x *Saccharomyces paradoxus* hybrids promote and mediate the occurrence of new LOH [49]. Hence, a plausible scenario is the occurrence of an increased recombination rate in the extremities of *C. orthopsilosis* chromosomes, similar to what has been described for *C. albicans* [42], leading to the overlap of recombination events, and consequent increased size of the LOH region, compatible with the expected increased meiotic recombination rate at the ends of the chromosomes [47]. Nevertheless, by itself, this finding is not sufficient to claim the existence of meiotic recombination in *C. orthopsilosis*, as the enrichment of LOH blocks in chromosome extremities that we observed in our UV experiments could also explain the patterns of natural *Candida* hybrids in a scenario of stress-induced DNA breaks (Table 1).

Previous studies have reported a positive correlation between the number of meiotic recombination events and chromosome size in *S. cerevisiae* and *Lachancea kluyveri* [50, 51], which is at odds with our observations for break-induced DNA repair where most events occurred in smaller chromosomes (Additional file 10, Table 1). We tested whether chromosome size correlated with the number or size of LOH events for all *C. orthopsilosis* isolates. Similarly to what was previously observed for MCO456 [16], our results revealed a statistically significant negative correlation of LOH block size and a positive correlation of number of blocks with chromosome size for all strains of clade 1, except s1799, which presented similar trends, but was not statistically significant (Fig. 4c, d, Table 1). Besides clade 1, the strain s498 also presented a significant negative correlation between LOH block size and chromosome size and positive correlation between the number of LOH events and chromosome size (Table 2). However, given the possibility of overlap of LOH events, we consider that these trends are compatible with but inconclusive with respect to their origin from meiotic recombination.

**Table 2:** Results of the Spearman correlation test between the average number/size of LOH and chromosome size for the strains of *C. orthopsilosis* clade 1 and the strain s498. Spearman rho is indicated for each comparison, and the respective *p*-value is between parentheses

Strain	LOH size vs chromosome size	LOH number vs. chromosome size
MCO456	− 0.7619048 (0.03676)	0.7619048 (0.03676)
s1799	− 0.6428571 (0.09618)	0.7142857 (0.05759)
s423	− 0.7619048 (0.03676)	0.7857143 (0.02793)
s434	− 0.7619048 (0.03676)	0.7619048 (0.03676)
s498	− 0.7857143 (0.02793)	0.7619048 (0.03676)

We set out to test this hypothesis experimentally following the same approach as Guitard et al., who reported sporulation in a *C. inconspicua* strain which was later proven to be a hybrid [13, 52] (see the “Methods” section). To do so, we used the MCO457 strain, which has both mating-type idiomorphs. We assessed the presence of spores by microscopy techniques [52], sequenced the genome of the same strain after the experiment, and searched for additional LOH blocks when compared to the time 0. Our attempts to induce sporulation were, however, unsuccessful and no differences were found at the genomic level, possibly indicating an inability to undergo meiosis. Similarly to what was previously described for other *Candida* species of the CUG-Ser clade [41], essential components for the occurrence of meiosis are absent in *C. orthopsilosis* and *C. metapsilosis* genomes (Additional file 16). Nevertheless, these genes are also absent in sexual *Candida* lineages [41], and thus, *C. orthopsilosis* and *C. metapsilosis* have in principle the necessary meiosis toolkit for the clade. Therefore, an alternative explanation for our unsuccessful results could be a failure to trigger meiosis in our experimental conditions.

## Discussion

Here, we analyzed the genomic aftermath of hybridization in the *C. parapsilosis* species complex, using improved assemblies and algorithms, and a comprehensive collection of 60 sequenced strains from at least six different hybridization events. This set includes 17 newly sequenced strains collected in an effort to find new hybridization events and the potential parental lineages of these hybrids. Three new strains of *C. orthopsilosis* were found to be homozygous and correspond to the already known parent A of these hybrids [18]. The absence of the unknown *C. orthopsilosis* parent B or any of the two unknown *C. metapsilosis* parents in the extended dataset reinforces the previously proposed idea that hybrids are more frequent among clinical isolates, which in turn could suggest a higher capacity to colonize and/or infect humans as compared to their homozygous parentals [1, 12, 13].

Much has been discussed about the origin of these hybrid strains, including whether it could have happened in the human host or in the environment. Our new sampling comprises the first sequenced environmental isolate of *C. metapsilosis*, from a marine source, which we prove to be a hybrid. Analysis of deposited ITS sequences from other environmental samples did not allow us to discard the possibility of contamination but suggests that members of the *C. parapsilosis* species complex can inhabit marine environments. Although not allowing an absolute conclusion about the origin of *C. metapsilosis*, this finding makes it more likely that the hybridization event occurred in the environment rather than in the host [1, 12]. Considering the increasing number of hybrid pathogens being described [1, 12–16, 18–20, 22], this process should be regarded with particular concern in the context of climate change and globalization promoting the contact between divergent lineages [53].

Hybrid genomes evolve through LOH which results in distinct patterns that differentiate strains and hybrid clades. In this study, there are limitations associated with the sample size that may underpower our ability to detect selection. Nevertheless, even with these limitations, we found that the number of overlaps of homozygous and heterozygous regions among these hybrids is larger than what is expected to occur by chance, and we found an extreme example of two genes that were always heterozygous not only in all the hybrid strains of the *C. parapsilosis* species complex, but also in hybrids of the distantly related *C. inconspicua*. Moreover, our analyses revealed that strains with similar nuclear and mitochondrial backgrounds at their origin (clades 1 and 3 of *C. orthopsilosis*) followed a different LOH direction in some PPR genes (possibly associated to mito-nuclear incompatibilities), suggesting that the absence of high heterozygosity, but not the retention of a specific haplotype, is the target of selection. A possible explanation to this trend could be the dimerization of some of these proteins, which could be affected by heterozygosity (i.e., less stable heterodimers) but not by homozygosity regardless of the parent favored by LOH. Still, we found very few instances of functional enrichment in nuclear genes preferentially covered by LOH regions. In the case of *C. metapsilosis*, considering very relaxed thresholds, we found that cell-wall composition might have been under selection for reducing heterozygosity. We speculate that perhaps the adaptation to a new environment (like the human body) could select for homozygosity in cell-wall composition genes. However, this result should be taken with caution, as statistical significance disappeared when considering more strict thresholds. Altogether, these results indicate that, although we found some evidence for Bateson-Dobzhansky-Muller incompatibilities, they are apparently not widespread or too weak to drive genome-wide convergent functional patterns in independently formed hybrids, and thus, genetic drift might be the prevailing force shaping LOH patterns. A lack of overall strong selection for homozygosity is in agreement with the absence of fitness differences between evolved strains that underwent LOH and their unevolved parents. In addition, it is important to remember that, in the presence of non-selective constraints (such as regions prone to recombination), non-random distributions of LOH across genes might also emerge. Alternatively, functional enrichment tests are not sufficiently fine-grained to detect the target of selection, or characteristics other than the function of the genes might be the target of selection. In this respect, a recent study found that genes with allele-specific expression were less likely to undergo LOH in *C. orthopsilosis* strains [54], which can suggest that selection at the transcriptional level might be playing a role in retaining heterozygosity. Further transcriptomic analysis using a larger sample size might help to further clarify hybrid genome evolution.

**Fig. 5:**
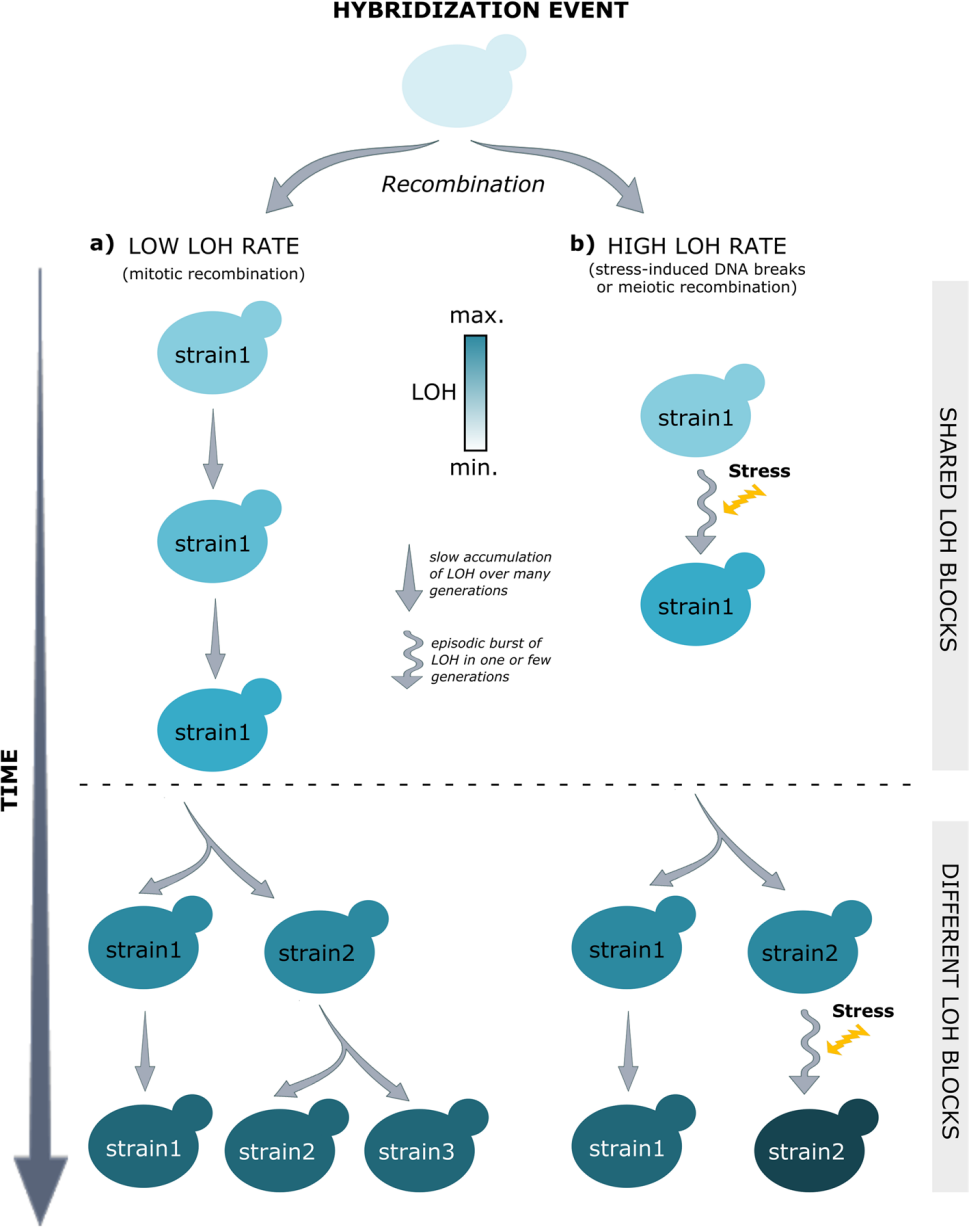
Schematic representation of the expected levels of LOH acquisition through **a** mitotic recombination or **b** stress-induced DNA breaks or meiotic recombination. The color grade scale indicates the color variation between low levels of LOH (light blue) and high levels of LOH (dark blue). The dashed line marks the common ancestor of strains of the same clade, after which different strains of a clade start diverging. In a scenario where only mitotic recombination takes place, the acquisition of LOH blocks is expected to proceed slowly and be proportional to time. In addition, SNPs and LOH levels are expected to increase in parallel as both processes would linearly accumulate with time. In a scenario of stress-induced DNA breaks or meiotic recombination, multiple LOH events are created in a single event, and strains progress much faster to high levels of LOH, as compared to mitotic recombination. In addition, LOH and SNP accumulation are uncoupled, and thus, levels of LOH and SNPs are not expected to correlate

The balance between selection and drift is influenced by different factors, including the size of the population, and the mechanisms by which genetic variability appears. Small population sizes and the existence of strong population bottlenecks limit the power of selection and favor genetic drift. We consider that this might have been an important factor contributing to the apparent weak signal for selection found in LOH patterns across *C. parapsilosis* hybrids. In addition, selection acts on entire genotypes, and we consider that the nature of how LOH patterns are generated, in contrast to point mutations, also limits the power of selection to favor homozygosity at particular loci. In fact, a single LOH event can affect several genes and dozens of heterozygous loci at the same time, and the advantage of homozygosity at certain loci might be compensated by deleterious effects of losing some alleles at other loci. Even if the selection does efficiently select particular homozygous loci, our ability to detect this selection will be hampered by the fact that this will be accompanied by many “passenger” genes that concomitantly become homozygous. These effects would be exacerbated if LOH occurs in an episodic manner, affecting multiple, scattered genomic areas in a short period of time. Our results indeed support an episodic nature of widespread LOH accumulation. Firstly, the finding of no single LOH difference in independent isolates from the same strain maintained at different labs suggests that LOH patterns are stable, at least over hundreds of mitotic divisions. This is supported by our results of in vitro evolved strains, which accumulated very few LOH events. Secondly, contrary to the expectation for a constant rate of accumulation of LOH through mitotic divisions, we found no correlation between the accumulation of LOH and the accumulation of point mutations in natural hybrid strains. These findings are compatible with an episodic nature of LOH accumulation, with periods of widespread LOH followed by long periods of stasis. We thus favor scenarios in which episodic events would generate multiple LOH events in one or few generations as opposed to scenarios in which LOH events accumulate slowly in a linear fashion (Fig. 5).

A previous study showed that the occurrence of LOH is often related to stress response [55], so we hypothesize that stress is one of the triggers of LOH in these hybrids and a major factor influencing their genomic features. This would explain the absence of new LOH events in strains kept for years in the lab. Our experiments suggest that the induction of DNA breaks by UV promotes LOH, effectively accelerating the rate of LOH accumulation. Although the UV-induced LOH events seem to be very restricted in number, we consider that, in scenarios of continuous stress, the increased rate of LOH formation could lead to the observed patterns in naturally occurring hybrid strains (Table 1). Additionally, our observations led us to consider the existence of meiotic recombination, at least in some *C. orthopsilosis* strains, as previously proposed [16]. First of all, the high amount of LOH blocks detected in the hybrids, together with the high similarity between strains of the same clade and the abovementioned apparent non-linearity of their accumulation, is highly suggestive that recombination between inter-hom(e)ologous chromosomes might be induced by meiosis. As meiosis is generally induced by stress, this idea is compatible with the stress-induced LOH hypothesis mentioned above. In support of meiotic breaks having a role in the formation of LOH, we found, in some strains, correlations between the length of the chromosomes and the size of LOH blocks, which would be unexpected from sporadic mitotic events and stress-induced DNA breaks. However, these results are still inconclusive as we are possibly underestimating the number of events and overestimating their respective size due to the possible overlap of LOH events. Moreover, similarly to previous experimental attempts [16, 41], we failed to induce sporulation in the lab.

Recently, alternative mechanisms that promote inter-hom(e)ologous chromosome recombination at a rate that is higher than what is expected for mitosis have been described. One such mechanisms is parameiosis [56], which was recently described in the opportunistic pathogen *C. albicans* (shown to be an evolved hybrid [14]). After parasex (mating of two *C. albicans* diploid cells) the tetraploid progeny tries to restore the diploid state through concerted chromosome loss [14, 57, 58]. This last process is what represents parameiosis, because it involves genes that are similar to those involved in meiotic recombination, and recombination occurs at very high rates [56]. Furthermore, this process seems to generate patterns of recombination compatible with the positive correlation between the number of recombination events and chromosome size that we observed for *C. orthopsilosis* hybrids [56]. Considering the failure to induce sporulation in *C. orthopsilosis*, we first hypothesized that parameiosis could be the underlying process of the LOH patterns we observed. However, such a mechanism would imply multiple rounds of polyploidization followed by chromosome loss. In such a scenario, we would not expect hybrids from the same clade to have so similar patterns. Based on this, we consider that it is improbable that parameiosis occurs in *C. orthopsilosis* hybrids.

Another possible mechanism could be the return to growth (RTG) after incomplete meiosis. This mechanism was shown for *S. cerevisiae* hybrids, which are able to enter meiosis, experience genomic recombination and return to growth without progressing to sporulation [59, 60]. RTG and meiosis involve inter-homeologous chromosome recombination with a similar rate but differ in the way molecule junctions are solved [46, 59]. This mechanism could explain the high amount of recombination events detected in *C. orthopsilosis* hybrids without the need of sporulation and would reconcile our genomic and experimental findings. Thus, despite our unsuccessful attempts to experimentally induce sporulation in these hybrids, we speculate that this or a similar process could be, together with stress-induced DNA breaks, responsible for the observed genomic patterns in *C. orthopsilosis*, and possibly in *C. metapsilosis* as well. However, additional studies should be carried out to confirm or refute this hypothesis, and further clarify the mechanisms underlying LOH in *Candida* hybrids.

## Methods

### Genomic DNA sequencing

Genomic DNA extraction of *C. orthopsilosis* and *C. metapsilosis* strains was performed using the MasterPure Yeast DNA Purification Kit (Epicentre) following the manufacturer’s instructions with some modifications. Briefly, *C. orthopsilosis* and *C. metapsilosis* cultures were grown in an orbital shaker overnight (200 rpm, 30 °C) in 15 ml of YPD (Yeast Extract–Peptone–Dextrose) medium. Cells were harvested using 4.5 ml of each culture by centrifugation at maximum speed for 2 min, and then they were lysed at 65 °C for 15 min with 300 µl of yeast cell lysis solution (containing 1 µl of RNAse A). After being on ice for 5 min, 150 µl of MPC protein precipitation reagent was added to the samples, they were centrifuged at 16.000* g* for 10 min to pellet the cellular debris, and the supernatant was transferred to a new tube. In the case of samples used for Illumina sequencing, DNA was directly precipitated by adding cold 100% ethanol, leaving the sample for 2 h at − 20 °C and centrifuging them at 16.000* g* for 30 min at 4 °C. The pellet was washed in 70% ethanol and left to dry. TE buffer was used to resuspend the DNA. The Genomic DNA Clean & Concentrator kit (ZymoResearch) was used for the final purification. For the sample used to obtain long reads (*C. metapsilosis* BP57 strain), 10 µl of RNase A/T1 mix (Thermo Fischer Scientific) was added to the recovered supernatant, and the sample was incubated at 37 °C for 2 h to ensure the complete removal of RNA. Then a phenol–chloroform purification step followed by a chloroform purification was performed using PLG (Phase Lock Gel) Heavy tubes: after 30 s of centrifugation of the PLG tubes to pellet the gel, first the sample and then an equal volume of phenol–chloroform were added into the tube, vortexed and centrifuged at 16.000* g* for 5 min. Then, an equal volume of chloroform was added into the same tube, vortexed, and centrifuged again at 16.000* g* for 5 min. The final aqueous phase was recovered into a new tube, where DNA was precipitated using 100% cold ethanol and centrifuging the samples at 16.000* g* for 30 min at 4 °C. The pellet was washed twice with 70% cold ethanol and, once the pellet was dried, the sample was resuspended in 50 µl of TE.

Illumina whole-genome sequencing was performed at the Genomics Unit from the Center for Genomic Regulation (CRG). As the samples were not sequenced all at the same time, but rather in two groups at different timepoints (group 1: CBS10746, CBS10747, CBS11127, CBS2916, SZMC21155, SZMC8099, and Ch_T3; group 2: 109, 172, 85, 88, 89, IFM48364, IFM48386, MCO457, MCO471), the protocol was not always the same. Differences in the protocol are mentioned across the method description. Illumina libraries were prepared using the NEBNext® DNA Library Prep Reagent Set for Illumina® kit (New England BioLabs) according to the manufacturer’s protocol. Briefly, 1 µg of gDNA was fragmented by nebulization in Covaris to approximately 600 bp and subjected to end repair, addition of “A” bases to 3′ ends and ligation of Truseq adapters. All purification steps were performed using Qiagen PCR purification columns (Qiagen) for group 1 and AMPure XP beads (Beckman Coulter) for group 2. Library size selection was done with 2% low-range agarose gels. Fragments with an average insert size of 700 bp (for group 1) and 665 bp (for group 2) were cut from the gel, and DNA was extracted using a QIAquick Gel extraction kit (Qiagen) and eluted in 30 µl EB; 10 µl of adapter-ligated size-selected DNA was used for library amplification by PCR using the Truseq Illumina primers. Final libraries were analyzed using Agilent DNA 1000 chip to estimate the quantity and check size distribution and were then quantified by qPCR using the KAPA Library Quantification Kit (KapaBiosystems, ref. KK4835) prior to amplification with Illumina’s cBot. Libraries were loaded at a concentration of 2 pM onto the flow cell and were sequenced 2 × 125 bp on Illumina’s HiSeq 2500.

Whole-genome sequencing of long reads of *C. metapsilosis* BP57 was performed at the CNAG-CRG (Centre Nacional d’Anàlisi Genòmica—Centre de Regulació Genòmica) using Oxford Nanopore Technologies (ONT). Genomic DNA was quality controlled on pulse field electrophoresis gel (Pippin Pulse, Sage Science) for DNA integrity, and the 260/280 and 260/230 ratios were used to detect the contamination on DNA samples with NanoDrop Spectrophotometer (Thermo Fisher Scientific). Then, the sample was used to prepare one 1D genomic library using the Ligation sequencing kit SQK-LSK108 for sequencing on a MinION instrument (Oxford Nanopore Technologies, ONT); 2 μg of genomic DNA were nick-repaired using the NEBNext FFPE DNA Repair Mix (NEB, M6630) and purified with 0.4X Agencourt AMPure XP Beads (Beckman Coulter, A63882). The samples were end-repaired and dA-tailed using the NEBNext UltraII End Repair/dA-Tailing Module (NEB, E7546) and subsequently purified with 1X Agencourt AMPure XP Beads. Then the 1D sequencing Adapter Mix (AMX1D, ONT) was ligated to the purified sample using the Blunt/TA Ligase Master Mix (NEB, M0367L). The adapter-ligated product was purified using 0.4-fold excess of AMPure XP beads. The beads were washed twice using the Adapter Bead Binding Buffer (ABB, ONT) and the libraries were eluted in 15 μl of Elution Buffer (ELB, ONT). The final library was loaded into R9.4 chemistry FLO-MIN106 flow cells (ONT) according to the manufacturer’s recommendations. In brief, firstly the MinKNOW interface QC (ONT) was run in order to assess the flow cell quality, and it was followed by the flow cell priming. The sequencing library was mixed with running buffer, Library Loading Beads (ONT), and nuclease-free water and loaded onto a “spot on” port for sequencing. The sequencing data was collected for 48 h. The quality parameters of the sequencing run were further monitored by the MinKNOW platform while the run was base-called using the Albacore 2.3.1.

Raw sequencing reads generated for this study can be found in SRA under the BioProject number PRJNA520893. The remaining genome sequencing libraries used for this work were retrieved from the BioProjects PRJEB4430, PRJEB1698, PRJNA322245, PRJNA579121, and PRJNA730502 [12, 16, 18, 21, 33].

### Genome assembly of *C. metapsilosis*

To obtain a better genome assembly for *C. metapsilosis*, we used a pipeline that combines both short and long-read assemblers to assemble sequencing data of the strain BP57 [61]. Briefly, BP57 Illumina reads were filtered and trimmed with Trimmomatic v0.36 [62] and assembled with Platanus v1.2.4 [63]. Nanopore reads were corrected with Canu [64] and assembled with DBG2OLC (v20180222) [65] using Platanus assembly, MaSurCA v3.3.0 [66], and WTDBG2 v2.1 [67]. Ragout v2.2 [68] was used for scaffolding using DBG2OLC, WTDBG2, and MaSurCA assemblies. Assembly correction was performed with Pilon v1.22 [69]. The quality of each of the assemblies was assessed with Quast v4.5 [70] and K-mer Analysis Toolkit v2.4.1 (KAT, [71]). In the end, DBG2OLC assembly after Ragout was the one with the best results and was used for downstream analysis (details in the “Results” section and Additional file 1). Augustus v3.5 [72] was used for genome annotation, using *C. albicans* as model species. Assembly completeness was assessed with BUSCO v3 [73].

### Read mapping and variant calling

All paired-end Illumina libraries of the 19 *C. metapsilosis* strains and 41 *C. orthopsilosis* strains (Additional file 24: Table S1) were inspected with FastQC v0.11.5 (http://www.bioinformatics.babraham.ac.uk/projects/fastqc/) and trimmed and filtered with Trimmomatic v0.36 [62]. Read mapping and variant calling were performed using HaploTypo v1.0.1 considering default parameters [74]. Briefly, filtered reads were mapped on the respective genome references (*C. metapsilosis* BP57 assembly and *C. orthopsilosis* 90–125 assembly [34]) with BWA-MEM v0.7.15 [75]. GATK v4.0.2.1 [76, 77] was used for variant calling using HaplotypeCaller and VariantFiltration tools, requiring a minimum read depth of 30 reads per position. The mapping was inspected with IGV version 2.0.30 [77]. Mapping coverage was determined with SAMtools v1.9 [78]. A similar approach was used to align the genomic reads on the respective reference mitochondrial genome (accession numbers: JQ062879.1 for *C. metapsilosis* and NC_006972.1 for *C. orthopsilosis*). It is important to mention that in the case of the recently sequenced isolates described by [33], multiple sequencing data was available for *C. metapsilosis* and *C. orthopsilosis*. However, as confirmed by the phylogenies provided by the same authors, all of them corresponded to the same strain, and therefore, we have selected only one of each species to include in our dataset (MSK446 in the case of *C. metapsilosis* and MSK636 in the case of *C. orthopsilosis*). In order to determine the number of SNPs/kb, a file containing only SNPs was generated with the SelectVariants tool. Moreover, for this calculation, only positions in the reference with more than 30 reads were considered for the genome size, and these were determined with bedtools genomecov v2.25.0 [79].

### LOH blocks and gene homozygosity definition

Taking advantage of the presence of more than one library for the same strain in our dataset (s424), the reproducibility of the procedure for LOH block definition applied and validated by Pryszcz et al. was tested [12]. Briefly, we determined LOH blocks for the different libraries of the same strain by filtering out regions with < 0.75% and > 1.25% coverage (as previously applied), and not applying any coverage filter. The overlap of the LOH blocks was taken as a proxy for the reproducibility of the method. This overlap was determined with the Jaccard metric of bedtools v2.25.0 [79]. Our analysis indicated that the results were only reproducible removing the coverage filter (see Additional file 2). Thus, for LOH blocks definition, bedtools merge v2.25.0 [79] with a window of 100 bp was used to define heterozygous regions, and by opposite, LOH blocks would be all non-heterozygous regions in the genome. The minimum LOH block size was established at different thresholds, namely, 100 bp, 150 bp, 200 bp, 500 bp, 1 kb, and 5 kb, and therefore, 6 parallel analyses were performed. The “degree of homozygosity” per gene was defined as the number of positions in a given gene coinciding with LOH blocks per gene size, and it was calculated with bedtools coverage v2.25.0 [79]. The overlap of homozygous and heterozygous genes between strains or clades was performed with hypergeometric tests using the phyper function of R, setting lower.tail to false.

### Functional gene annotation

GO terms for *C. orthopsilosis* were retrieved from *Candida* Genome Database (http://www.candidagenome.org/) on September 27, 2019. To complement this information, functional annotation was performed for both species (*C. orthopsilosis* and *C. metapsilosis*) with InterProScan v5.21.6 [80], and EggNOG-mapper v1.0.3 using Diamond algorithm [81]. From this analysis, we retrieved information not only on GO terms, but also on KEGG pathways, Pfam domains, and orthologs.

### Enrichment analysis

To inspect if any function was preferentially undergoing LOH in the hybrid strains, for each hybridization event, the average and the median of homozygosity per gene was calculated. Enrichment analysis was performed comparing two different datasets, namely, heterozygous and homozygous genes using FatiGO [82] in order to find GO terms, KEGG terms, or Pfam domains over-expressed in one of the lists. However, to do that, it was necessary to determine a threshold between homozygous and heterozygous genes. Thus, this threshold was set at 100%, 90%, 80%, and 50% of the mean or median homozygosity for each gene. Moreover, as conserved genes could be influencing the results, we have decided to exclude from the analysis all the genes without non-synonymous SNPs. Analyses were performed considering only genes with at least one non-synonymous mutation in at least one strain, and later considering all the genes (Additional files 17, 18, 19, and 20). As we were also interested in assessing if any function was kept as heterozygous, a similar approach was performed for 0%, 10%, and 20% “gene homozygosity.”

### Pentatricopeptide repeat proteins

Due to their possible role in genomic incompatibilities, PPR proteins were a special focus of this work. PPR proteins were selected considering the list previously described for *S. cerevisiae* and *C. albicans* [83]. The orthologs of these genes were retrieved from the analysis with EggNOG-mapper v1.0.3 [81, 83]. A search for PPR domains in the remaining proteome was performed with HMMER (http://hmmer.org). Proteins predicted by functional gene annotation analysis as PPRs were also added to this dataset. In total, we analyzed 13 PPR proteins. The level of homozygosity was inspected for each gene in each strain. In case of genes 100% homozygous in all strains from a given clade, the respective genomic region was inspected with IGV version 2.0.30 [77].

### Determination of the different hybridization events

Previous authors have identified two hybridization events in the origin of *C. metapsilosis* and at least four independent hybridization events in the origin of *C. orthopsilosis* [12, 18, 21]. To check whether the new strains considered in this project belong to one of these hybridization events or to new ones, bedtools jaccard v2.25.0 [79] was used to quantify the number of nucleotide positions in the union of LOH blocks in strain pairwise comparisons.

Furthermore, for each *C. orthopsilosis* and *C. metapsilosis* strain, the homozygous variants were substituted in the respective reference genome with the FastaAlternateReferenceMaker tool of GATK v3.6 [76]. Positions with heterozygous variants or INDELs in at least one of the respective strains were removed from the final alignments with bedtools subtract v2.25.0 [79]. In the end, two concatenated alignments of 12,028,303 and 11,886,656 nucleotides were obtained for *C. orthopsilosis* and *C. metapsilosis* strains, respectively. A maximum likelihood tree representative of each alignment was generated with RAxML v8.2.8 software using the GTRCAT model [84]. A similar approach was applied to a concatenated alignment of the strains of *C. metapsilosis* clade 1 with 12,336,395 bp.

### Analysis of ITS sequences of environmental isolates

Publicly available ITS sequences for environmental isolates were retrieved from the NCBI database (LC415306.1, AB863470.1, AB863471.1, KJ194280.1, KJ194328.1, KJ194334.1, and KJ194336.1) [37–39]. After an online BLASTn of each of these sequences, we could separate them into two groups, as some corresponded to the 26S sequence (LC415306.1, AB863470.1, and AB863471.1) and the others to the ITS1 (KJ194280.1, KJ194328.1, KJ194334.1, and KJ194336.1). The ITS1 and 26S regions were retrieved for each C*. metapsilosis* strain after replacing the respective homozygous variants in the reference genome with the FastaAlternateReferenceMaker tool of GATK v3.6 [76]. Alignments were performed with MAFFT online interface [85, 86]. Positions with gaps were removed with trimAL v1.4.rev15 [87]. A phylogenetic tree representative of each alignment was generated with the RAxML v8.2.8 software using the GTRCAT model [84]. Of note, as in the ITS group of strains, there was an apparent long-branch attraction, the analysis was performed independently for each public sequence.

### Estimation of the divergence time for each hybridization event

To understand if the LOH level is directly correlated to the age of the different hybridization events, we compared the shared LOH blocks of all strains from a given clade and counted for each strain the number of SNPs which are not shared between all of them, assuming that in this case they represent mutations acquired after the divergence. To estimate the divergence time for each clade the average number of new SNPs per kilo-base per strain was considered. The estimated divergence time for each clade was compared with the average amount of LOH acquired after the clade divergence.

### Detection of chromosome recombination in heterozygous positions

To detect events of recombination within heterozygous blocks, we used an *in-house* script (https://github.com/Gabaldonlab/detect_recombination/blob/master/detect_cross_over_PHASING.py). Briefly, for each strain and each heterozygous block with more than 500 bp, we phased all the 0/1 heterozygous positions using HapCUT2 (release April 4, 2019, [88]). The average allele frequency of each block was calculated by dividing the number of reads supporting each haplotype in the heterozygous regions. All the blocks with ploidy different from 2 were excluded (allele frequency < 40% or allele frequency > 60%). We considered as potential regions of recombination, all the phased blocks with more than 10 consecutive SNPs supporting each of the possible phased genotypes (0|1 and 1|0). All the obtained results were inspected with IGV version 2.0.30 [77].

### *C. orthopsilosis* experimental assays

In order to investigate the mechanisms involved in the acquisition of LOH in *Candida* hybrids, isolates of *C. orthopsilosis* (strains 172, s424, and IFM48386) and *C. metapsilosis* (strain BP57) were plated from glycerol stock on YPD agar plate and left to grow for 2 days at 30 °C. All the following incubations were done at 30 °C. For the analysis of the changes obtained by the propagation of the samples in rich medium, single colony was replated every working day, 30 times on YPD agar plates. Furthermore, a single colony was selected and regrown on a liquid medium overnight from where the DNA for sequencing was extracted.

The intent to sporulate the MCO457 strain was performed by incubation of the streaked single colony on a medium containing yeast extract (0.25% w/v), glucose (0.10% w/v), potassium acetate (1% w/v), and agar (2% w/v) at 30 °C for 4 weeks (Guitard et al. 2015). Each week, a thin smear of the culture was stained using the Schaeffer and Fulton Spore Stain Kit (Sigma-Aldrich, cat. No 04551) following the producers’ protocol. A diploid *S. cerevisiae* was used as a positive control. Green/blue color indicating ascospores was seen in the positive control whereas no spores were observed in the investigated strain. For the detection of genomic modifications even without sporulation, and as an attempt to simulate a RTG process, a single colony was picked after 4 days of the initial incubation and plated on YPD media and left 3 days to grow. Furthermore, a single colony was selected and regrown on a liquid medium overnight from where the DNA for sequencing was extracted.

In the experiments in which cells were irradiated, the protocol was adapted from the previous work of Charles et al. [89] with few alterations. Briefly, an overnight sample was diluted to OD = 0.2 and left to grow for further 4 h. Cells were adjusted to OD = 0.1, pelleted, washed twice with water, and diluted in 1 ml of YPD medium. One hundred microliters of 100 × diluted sample was then distributed on a YPD agar plate and put under UV (254 nm) derived from a TL-2000 Ultraviolet Translinker at a dosage of 75 J/m^2^. Following the radiation, the plate was incubated for 2 days. Apart from regularly growing cells, small colonies were observed indicating slower growth. Single cells showing both phenotypes were regrown on YPD agar from where single colonies were selected and regrown overnight for DNA extraction used for sequencing.

### Fitness assays

The fitness of the evolved hybrid strains was assessed following the Q-PHAST protocol. Briefly, the strains under study were plated in rich YPD-agar medium for 48 h. Then, 4 independent CFUs were selected for each strain and inoculated in liquid YPD medium in different positions of a 96-deep well plate for 24 h. To perform the experiment, 3 μl of a 1/65 dilution of the saturated culture was inoculated in 4 plates of solid YPD medium. Therefore, a total of 16 replicates per strain (4 biological replicates with 4 technical replicates each, a total of 384 colonies to test the fitness) were made. These plates were incubated at 30 °C for 24 h on a scanner that took pictures every 15 min. Subsequently, the images were analyzed using Q-PHAST, where the growth of each of the colonies was measured during the course of the experiment and growth curves were made from which the area under the curve (AUC) was obtained. The nAUC of each strain was compared with that of its strain of origin obtaining the rel_AUC, the median (Median_rel_AUC), and the median absolute deviation (MAD_rel_AUC) were calculated.

## Supplementary Information

**Additional file 1.** Analysis of the available genome assemblies for *C. orthopsilosis* and C. metapsilosis.

**Additional file 2.** LOH inference provides a highly resolved map of genomic patterns across globally distributed hybrid strains.

**Additional file 3.** Summary of the variant calling and LOH blocks analyses for *C. metapsilosis* strains after read mapping on the reference genome. The different columns indicate various measured variables in this order, Strain: strain name or code; Other name: alternative strain names or codes; MAT: mating type; Type: hybrid or parental nature of the strain; Coverage: sequencing coverage; %reads mapped: percentage of sequencing reads mapped to the reference; Genome > 30 reads: number of positions in the reference genome mapped by more than 30 reads; Variants: number of called variants; PASS Variants: variants that pass the quality filters; PASS SNPs: variants that are single nucleotide polymorphisms; Heteroz Variant PASS: heterozygous variants; Homoz Variant PASS: homozygous variants; PASS Variants/kb; variant density; PASS SNPSs/kb: SNP density; Heteroz Variants/kb: heterozygous variant density; Homoz Variants/kb: homozygous variant density; LOH Blocks > 100bp: number of blocks larger than 100bp; LOH nucl >100bp: number of sites within blocks larger than 100bp; LOH genome >100bp: percentage of the genome sequence within LOH blocks larger than 100bp; Variants in Heter blocks: number of variants within heterozygous blocks; Heter nucl > 100bp: number of sites within heterozygous blocks larger than 100bp; Parental divergence: average nucleotide divergence between the two haplotypes in heterozygous blocks.

**Additional file 4.** Summary of the variant calling and LOH blocks analyses for *C. orthopsilosis* strains after read mapping on the reference genome. Indicated variables as in Additional file 3.

**Additional file 5.** Number of shared LOH blocks > 1 kb between all strains of the different clades.

**Additional file 6.** Gene overlap with LOH blocks and positive functional enrichment results obtained with indication of three different results. 1. Distribution of the frequency of gene homozygosity per strain in each of the hybrid clades. 2. Positive results of the GO enrichment analysis between genes covered in < 50% of their length by LOH blocksand genes covered in >= 50% of their length by LOH blocks, according to the mean of all C. metapsilosis strains, and using a minimum LOH block size of 100bp. 3. Positive results of the GO enrichment analysis between genes covered in < 50% of their length by LOH blocksand genes covered in >= 50% of their length by LOH blocks, according to the median of all C. metapsilosis strains, and using a minimum LOH block size of 100bp.

**Additional file 7.** *P*-values obtained from the hypergeometric test applied to compare the overlap of heterozygous and homozygous regions in each pair of strains or clades.

**Additional file 8.** List of the PPR proteins analyzed and respective level of homozygosity per strain.

**Additional file 9.** Mitochondrial inheritance and LOH in mtDNA-interacting pentatricopeptide repeat proteins.

**Additional file 10.** Results of the experimental test of the occurrence of LOH in *C. orthopsilosis* and *C. metapsilosis* during mitotic recombination and DNA-break induced repair. Pink, green and blue backgrounds highlight the occurrence of crossover, non-crossover and copy-number variation events, respectively.

**Additional file 11.** Summary of the variant calling and LOH blocks analyses for *C. orthopsilosis* strain s424.

**Additional file 12.** Results of the fitness assays of the evolved hybrid strains in comparison to their respective time 0.

**Additional file 13.** Detected non-reciprocal recombination events in *C. orthopsilosis*.

**Additional file 14.** Estimated clade divergence and acquired levels of LOH.

**Additional file 15.** Average percentage of LOH and number of recombination events in 1 kb windows of all *C. orthopsilosis* chromosomes, only considering the 16 randomly selected strains represented in Fig. 2b to avoid bias of different clade sampling size.

**Additional file 16.** List of essential genes for the occurrence of meiotic recombination, and their respective orthologs in *C. orthopsilosis*, *C. metapsilosis* and *Clavispora lusitaniae*, determined by EggNOG-mapper v1.0.3 using Diamond algorithm.

**Additional file 17.** *Candida metapsilosis* genes overlapping at least one non-synonymous mutation and respective GO terms used in the functional enrichment analysis.

**Additional file 18.** All *Candida metapsilosis* genes, independently of their overlap with non-synonymous mutations, and respective GO terms used later in the functional enrichment analysis.

**Additional file 19.** *Candida orthopsilosis* genes overlapping at least one non-synonymous mutation and respective GO terms used in the functional enrichment analysis.

**Additional file 20.** All *Candida orthopsilosis* genes, independently of their overlap with non-synonymous mutations, and respective GO terms used later in the functional enrichment analysis.

**Additional file 21: Fig. S1.** Pairwise comparisons in *C. orthopsilosis* and *C. metapsilosis* strains. The different hybrid clades are highlighted with the same colors as in Fig. 3.

**Additional file 22: Fig. S2.** Phylogenetic tree of the reconstructed ITS and 26S sequence alignment of all *C. metapsilosis* strains and publicly available sequences of environmental isolates. Sequences of environmental isolates retrieved from NCBI are highlighted in red, while the sequence of the environmental isolate sequenced in this study is highlighted in blue. Trees were rooted using *C. parapsilosis* as outgroup.

**Additional file 23: Fig. S3.** IGV screenshots of the read alignment of strains CBS 2916, CBS 10747 and CP367 in *C. metapsilosis* genome assembly. a) MAT locus region of *C. metapsilosis* genome assembly, where an LOH block is flanked by heterozygous regions. The different boundaries in the left side of the LOH block are highlighted with a red box. b) LOH blocks shared by clades 1.1 and 1.2 of *C. metapsilosis* covering the *MTC5* gene; c) LOH blocks shared by clades 1.1 and 1.2 of *C. metapsilosis* covering the *BPH1* gene.

**Additional file 24: Table S1.** Information relative to all *C. metapsilosis* and *C. orthopsilosis* strains used in this project, including strain’s name, type of organism, hybridization event, site and place of isolation, genome coverage, number of heterozygous variants per kilo-base, and the percentage of the genome with loss of heterozygosity considering a window of 100bp.

## Data Availability

The *C. metapsilosis* de novo genome assembly and all *C. orthopsilosis* and *C. metapsilosis* raw sequencing reads generated for this study can be found in NCBI under the BioProject PRJNA520893 [90]. *C. metapsilosis* genome annotation is also available in the GRYC database (http://gryc.inra.fr). Genomic variability in terms of SNPs of all studied samples is available at CandidaMine (http://candidamine.org/, Hafez et al. *in preparation*).
