# Supplementary material for: Evolution of loss of heterozygosity patterns in hybrid genomes of Candida yeast pathogens
Source: BMC Biol. 2023 May 11;21:105. doi: 10.1186/s12915-023-01608-z (PMC10173528; doi:10.1186/s12915-023-01608-z)
Supplement: Supplementary file 1 — Additional file 1. Analysis of the available genome assemblies for C. orthopsilosis and C. metapsilosis. [file 12915_2023_1608_MOESM1_ESM.docx]

**Supplementary file 1. Analysis of the available genome assemblies for *C. orthopsilosis* and *C. metapsilosis***

Recently, new genome assemblies were made available for both *C. orthopsilosis* and *C. metapsilosis* [[1, 2]](https://paperpile.com/c/Nb27Ao/0geK+znEj). As by the time of the release of this data, our analyses were almost complete, we decided to assess whether changing the reference genomes we were using by the new ones would improve our results.

In the case of *C. orthopsilosis,* the reference genome assembly available at NCBI at the time we started the project and the new one represent the same strain (90-125, [[1, 3]](https://paperpile.com/c/Nb27Ao/0geK+oQ7mj)). According to Lombardi et al. (2019), the main difference between the two assemblies is the fact that theirs was generated using long-read sequencing technology, therefore decreasing the amount of Ns introduced in the assembly, and also extending repetitive regions [[1]](https://paperpile.com/c/Nb27Ao/0geK). However, it is important to note that their assembly is divided in 10 scaffolds, while Riccombeni et al. (2012) assembly is divided in 8 chromosomes. *K*-mer analysis [[4]](https://paperpile.com/c/Nb27Ao/m0T6I) reported 99.05% completeness for Lombardi et al. (2019) assembly and 99.18% completeness for Riccombeni et al. (2012), suggesting that the new one is not more complete (Fig. 1_S1). The alignment between the two assemblies using MUMmer v3 [[5]](https://paperpile.com/c/Nb27Ao/xrU4Y), also revealed a high similarity between them (Fig. 2_S1). Moreover, a similar proportion of reads of the strain 90-125 aligned in both assemblies (92% for Lombardi et al. (2019) assembly and 91.5% for the previous one). For all these reasons, we considered that using the new assembly would not have a significant impact in our analysis, and therefore we decided to continue using the reference genome generated by Riccombeni et al. (2012) for *C. orthopsilosis*.

**
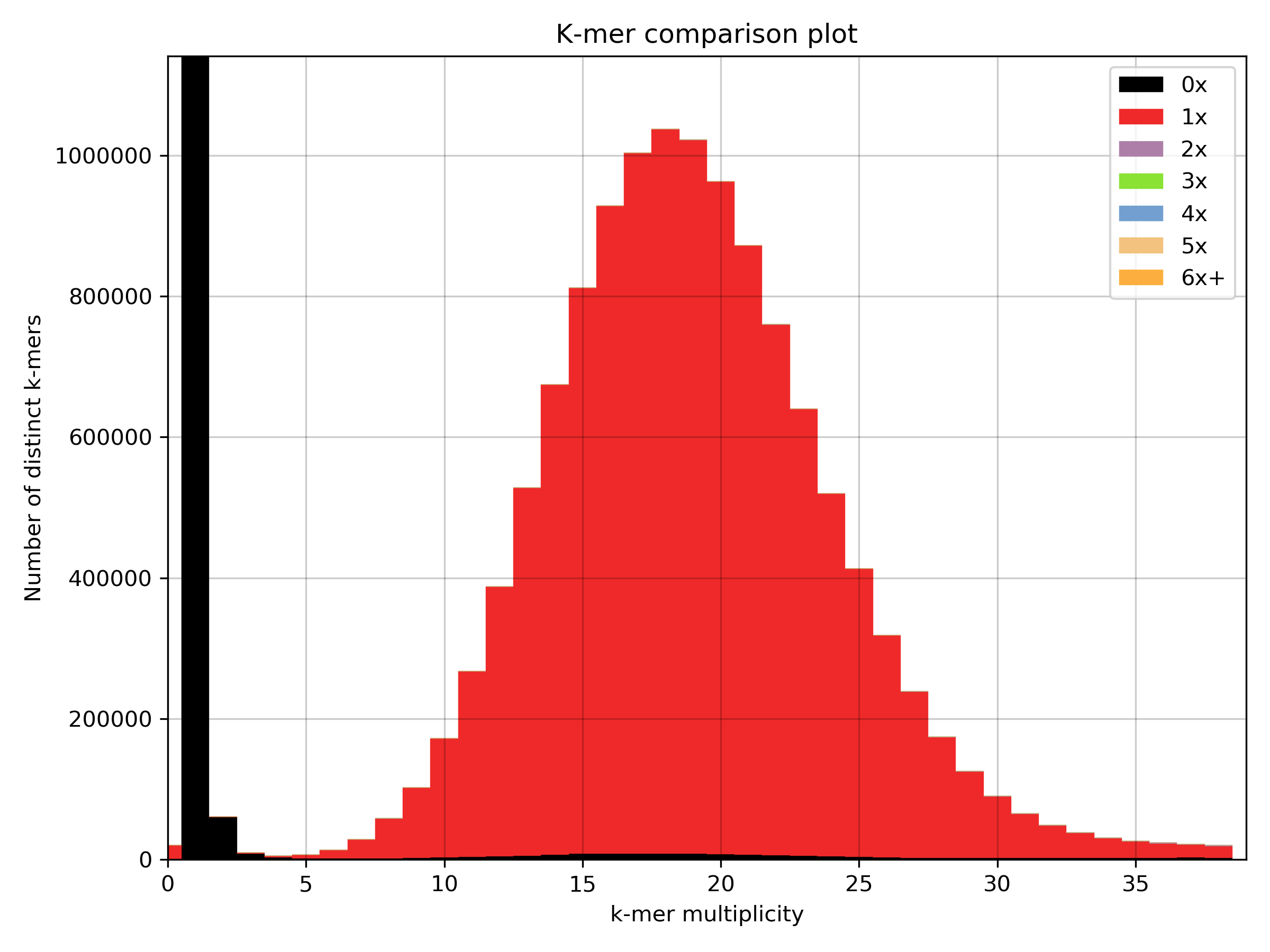

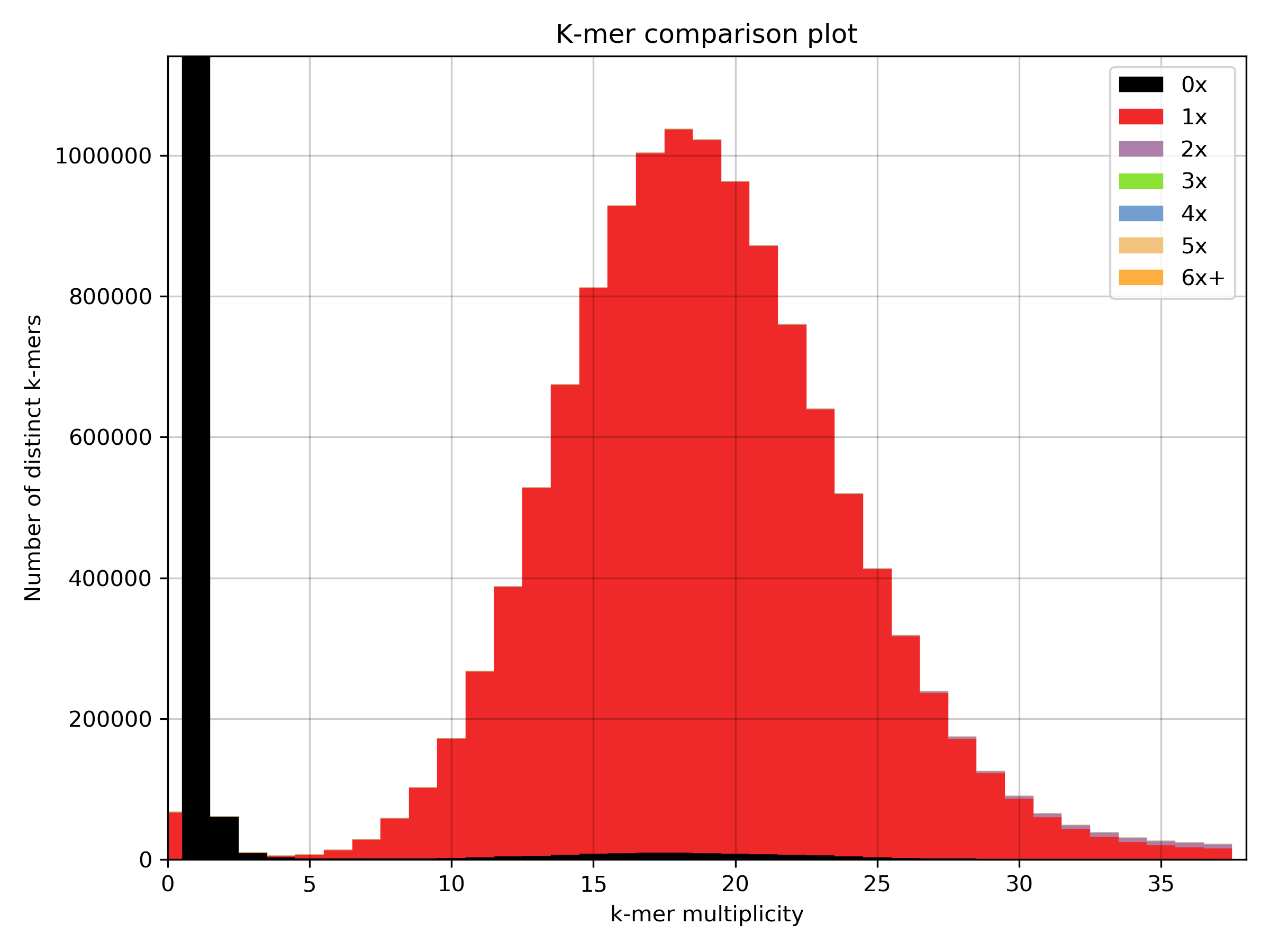
**

**Fig. 1_S1.** *K*-mer analysis of the assembly generated by Lombardi et al. 2019 (left) and Riccombeni et al. 2012 (right).

**
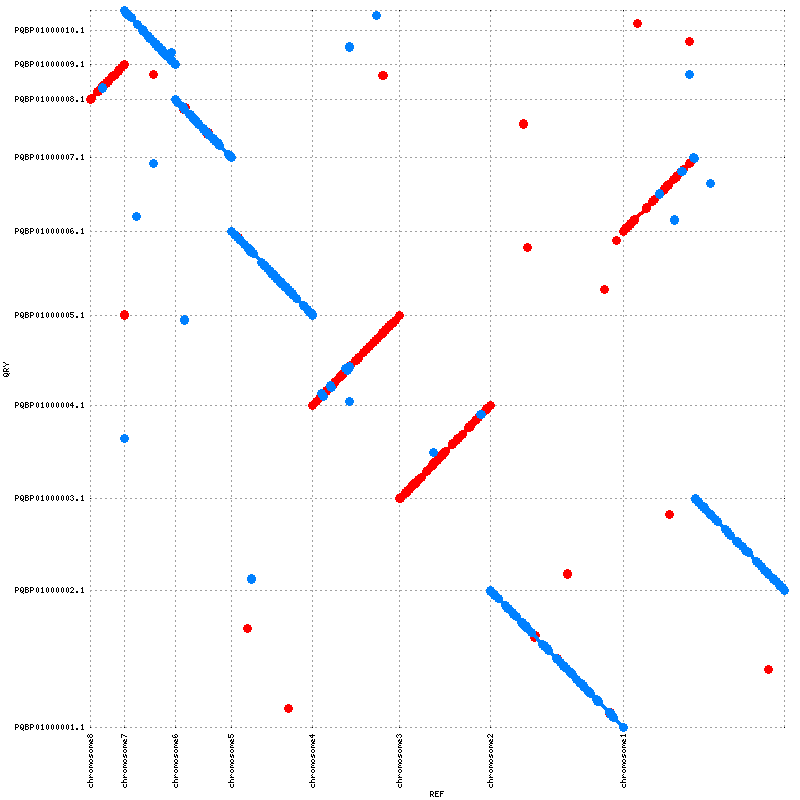
**

**Fig. 2_S1.** Alignment between Riccombeni et al. 2012 assembly (*x*-axis) and the assembly generated by Lombardi et al. 2019 (*y*-axis).

In the case of *C. metapsilosis*, at the time of the start of this project, the available genome assembly was fragmented (57 scaffolds) and chimeric (corresponded to two different strains) [[6]](https://paperpile.com/c/Nb27Ao/ohsTq). For this reason, we assembled a new genome using both long and short reads of the BP57 strain, “BP57 assembly”. Recently, Oh et al. (2019) published another assembly, the “ATCC_96143 assembly” [[2]](https://paperpile.com/c/Nb27Ao/znEj). While ATCC_96143 assembly comprises 13.6 Mb divided into 19 scaffolds, with an N50 of 3,752,469 bp, the BP57 assembly comprises 13 Mb divided into 9 scaffolds, with an N50 of 2,138,570 bp. Therefore, in terms of fragmentation, these last two assemblies are better than the first assembly of *C. metapsilosis*, and BP57 is better than ATCC_96143 assembly.

To compare the two less-fragmented assemblies, we performed their alignment using MUMmer v3 [[5]](https://paperpile.com/c/Nb27Ao/xrU4Y). As Fig. 3_S1 shows, their alignment did not reveal any significant difference, except for the presence of some short duplicated regions and a higher fragmentation in the ATCC_96143 genome assembly. This was confirmed by the similar amount of BP57 reads mapped to each of the assemblies (~93.5%). Unfortunately, *C. metapsilosis* strain ATCC_96143 that was used to assemble the ATCC_96143 genome has very low read coverage (2,014,500 reads, 60 reads/position), not allowing us to perform a proper *k*-mer comparison for assessment of assembly completeness. Therefore, we decided to assess this completeness using BP57 Illumina library. This allowed the confirmation that both ATCC_96143 and BP57 genome assemblies have a proper haplotype reduction (Fig. 4_S1), but in ATCC_96143 it appears that some of the homozygous regions are duplicated, which is consistent with the results obtained from the alignment of the two assemblies.


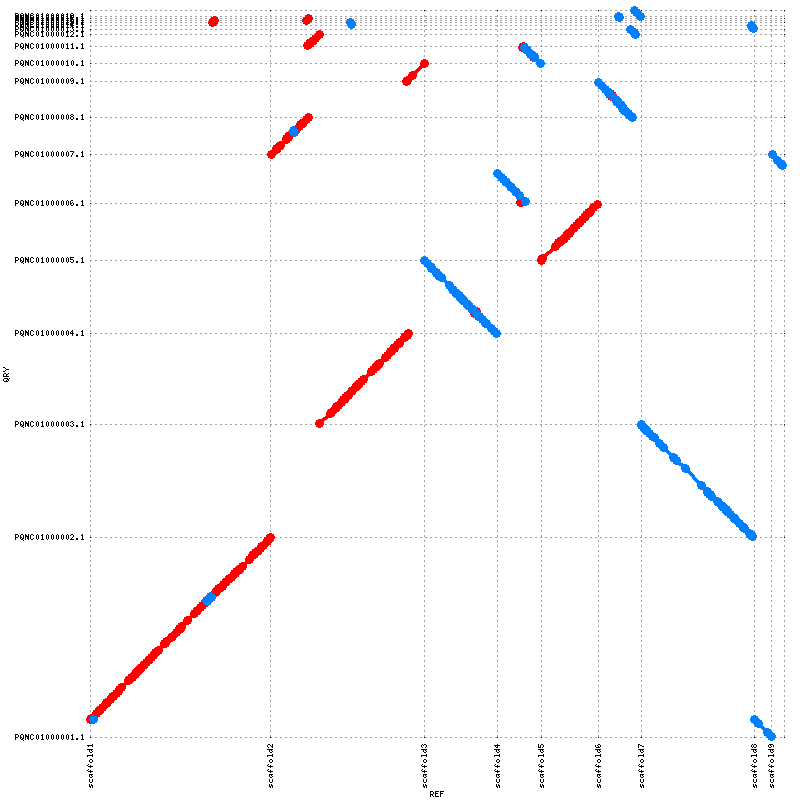


**Fig. 3_S1.** Alignment between BP57 assembly (*x*-axis) and the assembly generated by Oh et al. 2019 (*y*-axis).


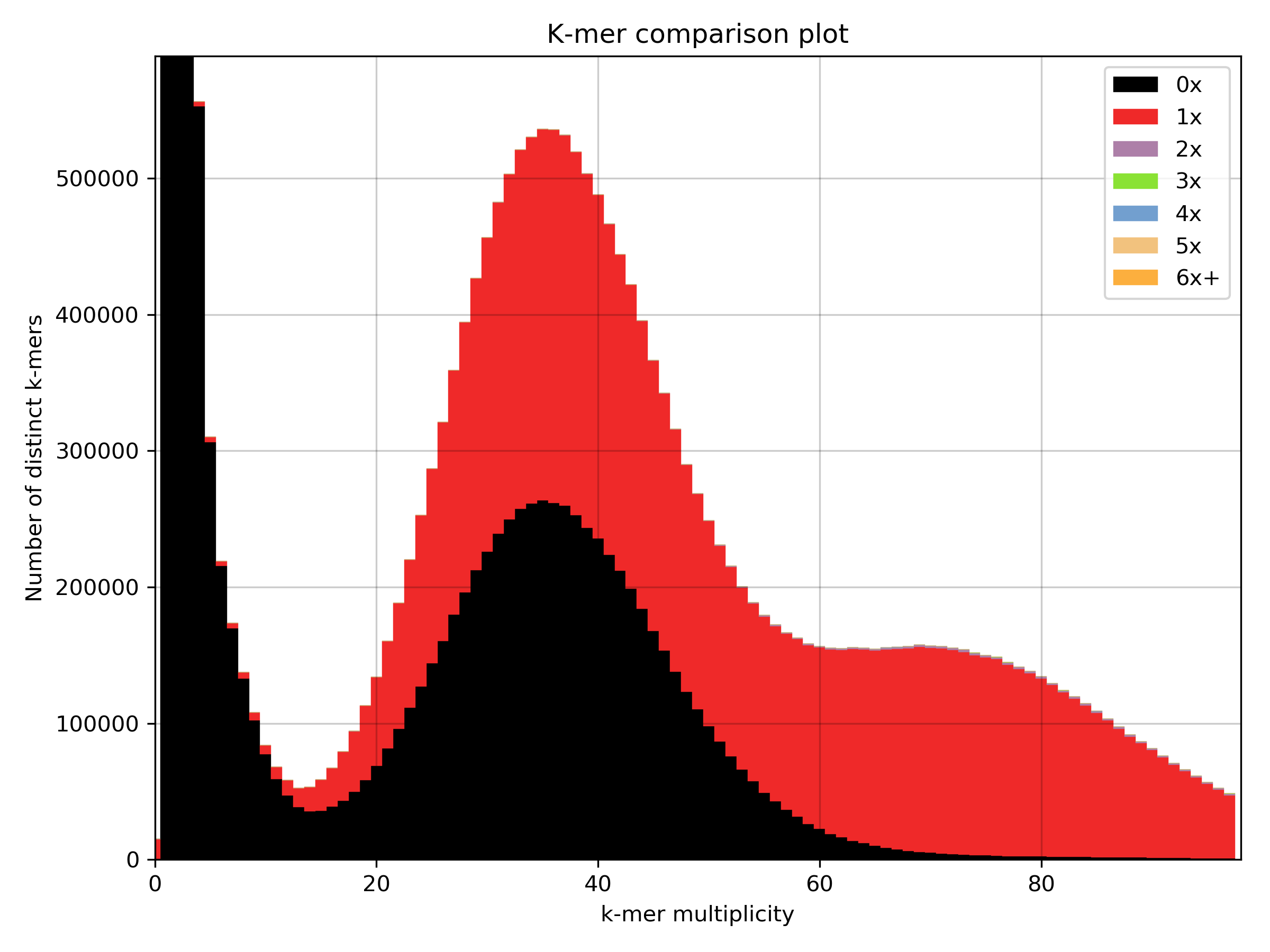

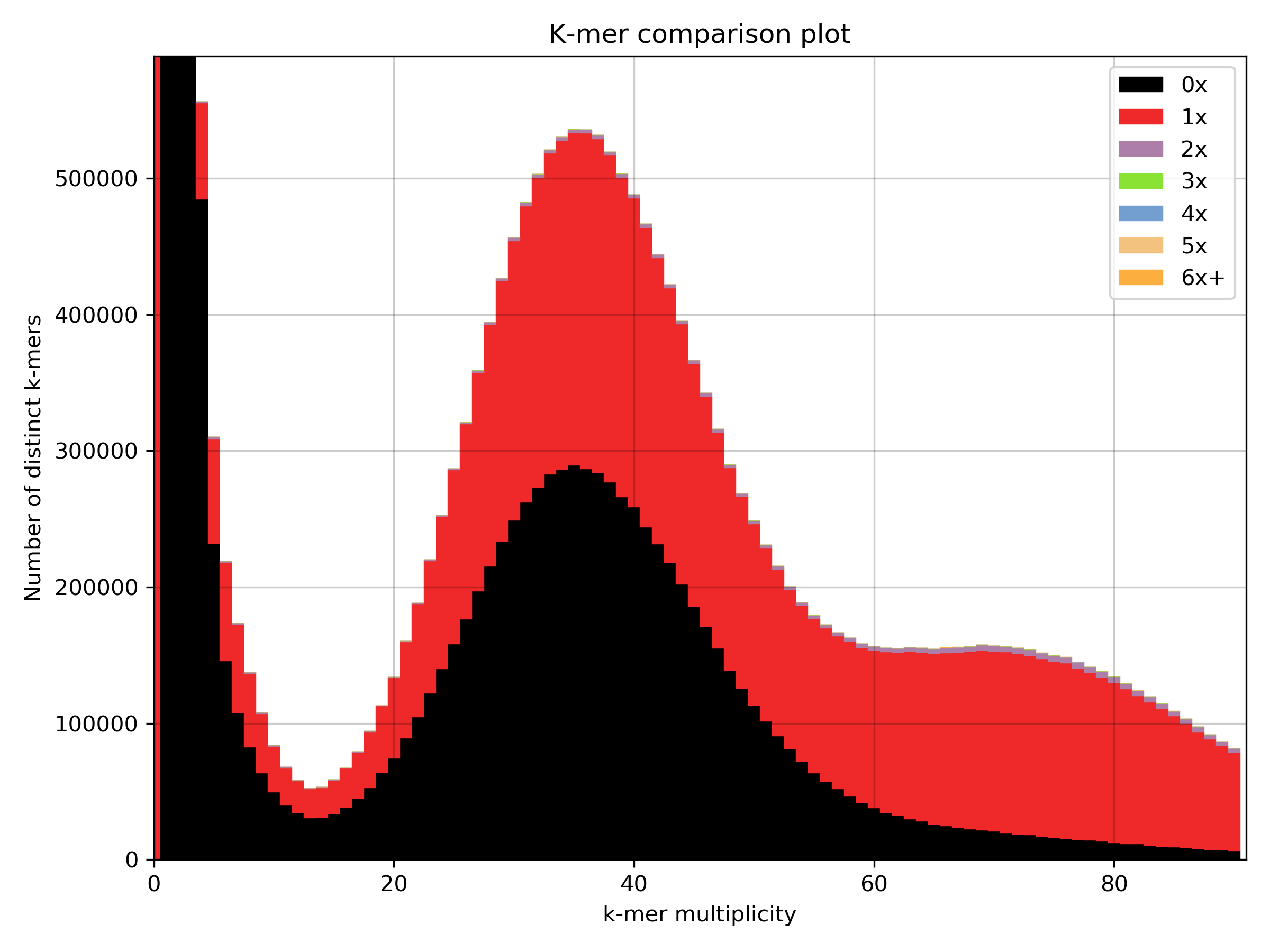


**Fig. 4_S1.** *K*-mer analysis of the BP57 genome assembly (left) and the ATCC_96143 genome assembly (right). Both assemblies have a proper reduction of the heterozygous regions.

In another attempt to estimate the assembly completeness, we performed the annotation of both ATCC_96143 and BP57 assemblies (see Material and Methods of main manuscript). Our analysis predicted 6,356 protein-coding genes for ATCC_96143 genome assembly, and 5,743 protein-coding genes for BP57 assembly. This last number is close to the number of distinct genes predicted in the previous chimeric assembly, 5,973 [[6]](https://paperpile.com/c/Nb27Ao/ohsTq), of which 5,440 are reciprocal best hits between the two (see Material and Methods of the main manuscript). Therefore, ATCC_96143 genome assembly had around 600 protein-coding genes more than expected. Trying to understand what was the cause of this difference, we identified 1,088 protein-coding genes in the ATCC_96143 assembly which did not have a mutual reciprocal hit in BP57 assembly. From these 1,088, only 60 did not have any hits. All the others corresponded to one of two situations: i) duplicated regions in genome assembly (which could be confirmed in Fig. 3_S1), and ii) split proteins. A random inspection of some of these split proteins revealed that for some reason, these genes in the ATCC_96143 assembly have multiple stop-codons in all the reading frames. We next assessed the quality of the assemblies with BUSCO v3 [[7]](https://paperpile.com/c/Nb27Ao/guAKp), which revealed that ATCC_96143 genome assembly is missing 7% of the proteins of Ascomycota database, as compared to 2.8% in BP57 genome assembly. Even more important, while only 2% of the proteins in BP57 genome assembly were found fragmented, in ATCC_96143 genome assembly this number increases to 21.7%, consistently with our analysis. For this reason, we considered the BP57 assembly the best assembly we had in hands. It is worth noting that, as our results depend on a proper variant calling step, and this depends on a good genome coverage, we have also decided to not include ATCC_96143 Illumina library in our analysis.

**References**

[1. Lombardi L, Zoppo M, Rizzato C, Bottai D, Hernandez AG, Hoyer LL, et al. Characterization of the Candida orthopsilosis agglutinin-like sequence (ALS) genes. PLoS One. 2019;14:e0215912.](http://paperpile.com/b/Nb27Ao/0geK)

[2. Oh S-H, Smith B, Miller AN, Staker B, Fields C, Hernandez A, et al. Agglutinin-Like Sequence () Genes in the Species Complex: Blurring the Boundaries Between Gene Families That Encode Cell-Wall Proteins. Front Microbiol. 2019;10:781.](http://paperpile.com/b/Nb27Ao/znEj)

[3. Riccombeni A, Vidanes G, Proux-Wéra E, Wolfe KH, Butler G. Sequence and analysis of the genome of the pathogenic yeast Candida orthopsilosis. PLoS One. 2012;7:e35750.](http://paperpile.com/b/Nb27Ao/oQ7mj)

[4. Mapleson D, Garcia Accinelli G, Kettleborough G, Wright J, Clavijo BJ. KAT: a K-mer analysis toolkit to quality control NGS datasets and genome assemblies. Bioinformatics. 2017;33:574–6.](http://paperpile.com/b/Nb27Ao/m0T6I)

[5. Kurtz S, Phillippy A, Delcher AL, Smoot M, Shumway M, Antonescu C, et al. Versatile and open software for comparing large genomes. Genome Biol. 2004;5:R12.](http://paperpile.com/b/Nb27Ao/xrU4Y)

[6. Pryszcz LP, Németh T, Saus E, Ksiezopolska E, Hegedűsová E, Nosek J, et al. The Genomic Aftermath of Hybridization in the Opportunistic Pathogen Candida metapsilosis. PLoS Genet. 2015;11:e1005626.](http://paperpile.com/b/Nb27Ao/ohsTq)

[7. Waterhouse RM, Seppey M, Simão FA, Manni M, Ioannidis P, Klioutchnikov G, et al. BUSCO Applications from Quality Assessments to Gene Prediction and Phylogenomics. Mol Biol Evol. 2018;35:543–8.](http://paperpile.com/b/Nb27Ao/guAKp)
