# Supplementary material for: Evolution of loss of heterozygosity patterns in hybrid genomes of Candida yeast pathogens
Source: BMC Biol. 2023 May 11;21:105. doi: 10.1186/s12915-023-01608-z (PMC10173528; doi:10.1186/s12915-023-01608-z)
Supplement: Supplementary file 2 — Additional file 2. LOH inference provides a highly resolved map of genomic patterns across globally distributed hybrid strains. [file 12915_2023_1608_MOESM2_ESM.docx]

**Supplementary file 2. LOH inference provides a highly resolved map of genomic patterns across globally distributed hybrid strains**

To assess genome evolution following hybridization we analyzed publicly available genomic data [[1–4]](https://paperpile.com/c/cHfmSL/S9Dk+rQdy+n0li+ms9A), and sequenced additional strains from *Candida parapsilosis* clade (see Material and Methods). The final dataset comprised 41 *C. orthopsilosis* and 19 *C. metapsilosis* strains, including the first sequenced *C. metapsilosis* strain isolated from an environmental source (Supplementary table 1). We analyzed these strains by mapping their genomic reads to their respective reference genomes and assessing genomic variations in terms of polymorphisms and LOH patterns (see Material and Methods). Our results show that all *C. metapsilosis* strains of clade 1, including the newly sequenced ones, are highly heterozygous, presenting a range of 21-32 heterozygous variants per kilo-base (kb, Supplementary table 1 and Supplementary file 3 for more details), while MSK414 (*C. metapsilosis* clade 2) has 42 heterozygous variants per kb. In the case of *C. orthopsilosis*, five strains are homozygous (90-125 and s428 as reported by [[1]](https://paperpile.com/c/cHfmSL/S9Dk), and 3 newly sequenced ones: 88, 89 and IFM48364), with an average of 0.1 heterozygous SNPs/kb. Consistent with the presence of different hybridization events [[1]](https://paperpile.com/c/cHfmSL/S9Dk), the remaining *C. orthopsilosis* strains showed a variable level of heterozygosity, ranging from 7 to 31 heterozygous variants per kb (Supplementary table 1 and Supplementary file 4 for more details). This confirms that, except for five *C. orthopsilosis* strains (90-125, s428, 88, 89 and IFM48364), all the strains are hybrids. Importantly, this includes the environmental strain of *C. metapsilosis* sequenced here (11127), suggesting that hybrids of this species can also exist in the environment.

We next compared patterns of LOH in these hybrid genomes. To refine our LOH inference methodology and test its reproducibility we here re-sequenced a highly heterozygous *C. orthopsilosis* strain (s424), which had been previously sequenced by another group [[1]](https://paperpile.com/c/cHfmSL/S9Dk). Considering the two sequencing libraries as replicates, we established a LOH block inference pipeline that ensured high reproducibility, independently of the minimum block size threshold considered (jaccard coefficient 0.964-0.989, depending on the minimum LOH-block size threshold), and improved over the previously used one (jaccard coefficient 0.641-0.706) [[2]](https://paperpile.com/c/cHfmSL/rQdy). Using this improved pipeline (see Material and Methods), we inferred LOH blocks in all hybrid strains. On average, considering a 100 bp minimum LOH block size, *C. metapsilosis* and *C. orthopsilosis* strains presented 11,384 and 6,916 LOH blocks, respectively (Supplementary files 3 and 4). Given the variable size of LOH blocks, their number is not indicative of the percentage of the genome in LOH regions. Thus, we calculated the total size of these blocks and found that on average 50.53% (range 28.91%-60.21%) of *C. metapsilosis* genome and 66.38% (34.19%-85.17%) of *C. orthopsilosis* genome underwent LOH, considering the less stringent threshold (Supplementary table 1 and Supplementary files 3 and 4). These values are similar between strains assigned to the same previously reported clades (Supplementary files 3 and 4), with *C. orthopsilosis* clade 1 being the most homozygous and clade 4 the most heterozygous one [[1]](https://paperpile.com/c/cHfmSL/S9Dk).

**References**

[1. Schröder MS, Martinez de San Vicente K, Prandini THR, Hammel S, Higgins DG, Bagagli E, et al. Multiple Origins of the Pathogenic Yeast Candida orthopsilosis by Separate Hybridizations between Two Parental Species. PLoS Genet. 2016;12:e1006404.](http://paperpile.com/b/cHfmSL/S9Dk)

[2. Pryszcz LP, Németh T, Saus E, Ksiezopolska E, Hegedűsová E, Nosek J, et al. The Genomic Aftermath of Hybridization in the Opportunistic Pathogen Candida metapsilosis. PLoS Genet. 2015;11:e1005626.](http://paperpile.com/b/cHfmSL/rQdy)

[3. Zhai B, Ola M, Rolling T, Tosini NL, Joshowitz S, Littmann ER, et al. High-resolution mycobiota analysis reveals dynamic intestinal translocation preceding invasive candidiasis. Nat Med. 2020;26:59–64.](http://paperpile.com/b/cHfmSL/n0li)

[4. O’Brien CE, Zhai B, Ola M, Bergin SA, Ó Cinnéide E, O’Connor Í, et al. Identification of a novel Candida metapsilosis isolate reveals multiple hybridization events. G3 . 2022;12.](http://paperpile.com/b/cHfmSL/ms9A)
