# Supplementary material for: Evolution of loss of heterozygosity patterns in hybrid genomes of Candida yeast pathogens
Source: BMC Biol. 2023 May 11;21:105. doi: 10.1186/s12915-023-01608-z (PMC10173528; doi:10.1186/s12915-023-01608-z)
Supplement: Supplementary file 6 — Additional file 6. Gene overlap with LOH blocks and positive functional enrichment results obtained with indication of three different results. 1. Distribution of the frequency of gene homozygosity per strain in each of the hybrid clades. 2. Positive results of the GO enrichment analysis between genes covered in < 50% of their length by LOH blocksand genes covered in >= 50% of their length by LOH blocks, according to the mean of all C. metapsilosis strains, and using a minimum LOH block size of 100bp. 3. Positive results of the GO enrichment analysis between genes covered in < 50% of their length by LOH blocksand genes covered in >= 50% of their length by LOH blocks, according to the median of all C. metapsilosis strains, and using a minimum LOH block size of 100bp. [file 12915_2023_1608_MOESM6_ESM.pdf]

Supplementary file 6. Gene overlap with LOH blocks and positive functional enrichment results obtained.

1. Distribution of the frequency of gene homozygosity per strain in each of the hybrid clades.

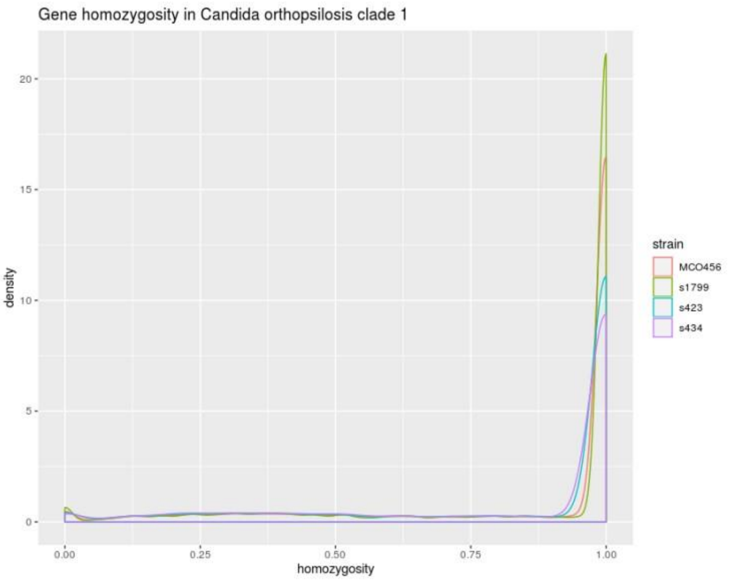

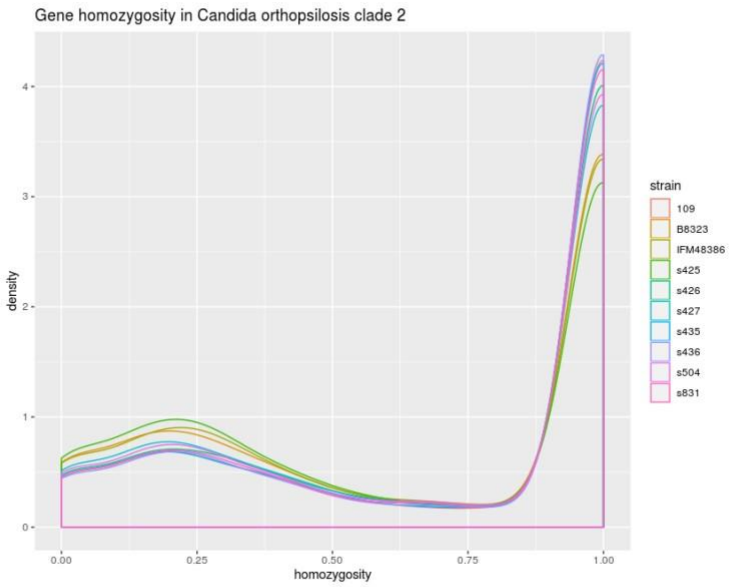

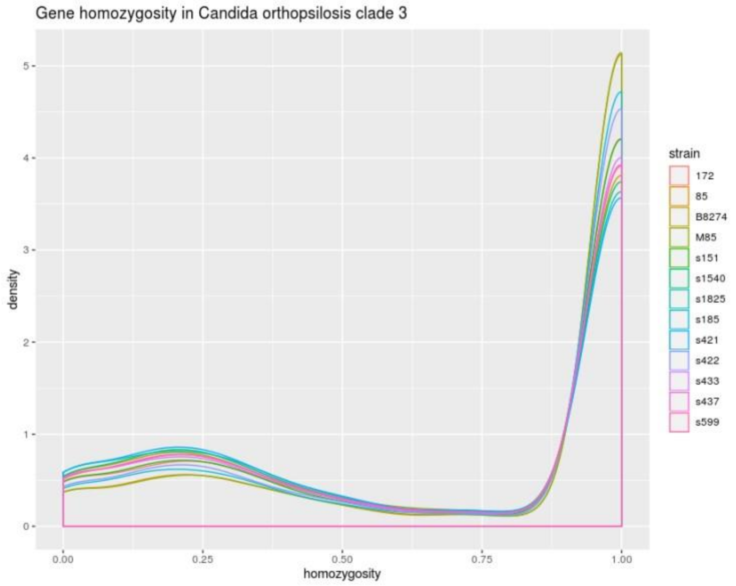

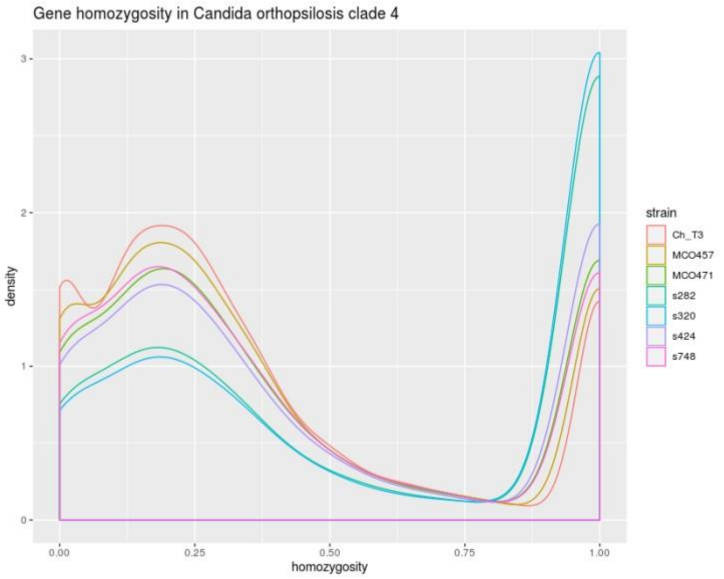

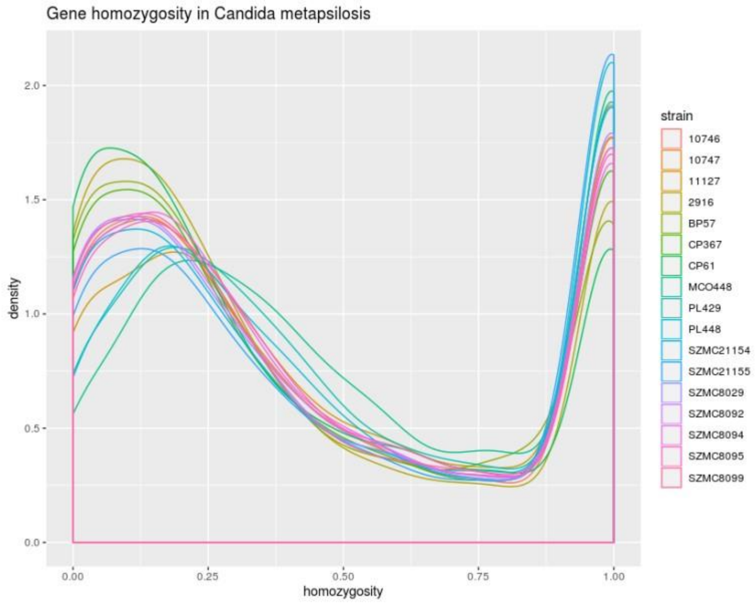

2. Positive results of the GO enrichment analysis between genes covered in < 50% of their length by LOH blocks (heterozygous) and genes covered in >= 50% of their length by LOH blocks (homozygous), according to the mean of all *C. metapsilosis* strains, and using a minimum LOH block size of 100bp.

Genes: 3716
Gos: 9893
Finished execution successfully.
Execution time: 1 sec.
\# OVER REPRESENTED TERMS
\# Term category: cellular_component

#overlist	term	term level	adj.pvalue	term name
1	GO:0005618	1	4.101590e-07	cell wall
1	GO:0009277	1	4.101590e-07	fungal-type cell wall
1	GO:0009986	1	3.707860e-06	cell surface
1	GO:0030312	1	4.101590e-07	external encapsulating structure

3. Positive results of the GO enrichment analysis between genes covered in < 50% of their length by LOH blocks (heterozygous) and genes covered in >= 50% of their length by LOH blocks (homozygous), according to the median of all *C. metapsilosis* strains, and using a minimum LOH block size of 100bp.

Genes: 3716
Gos: 9893
Finished execution successfully.
Execution time: 2 sec.
\# OVER REPRESENTED TERMS
\# Term category: cellular_component

#overlist	term	term level	adj.pvalue	term name
1	GO:0005618	1	1.249970e-04	cell wall
1	GO:0009277	1	1.249970e-04	fungal-type cell wall
1	GO:0009986	1	4.460300e-04	cell surface
1	GO:0030312	1	1.249970e-04	external encapsulating structure
