# Supplementary material for: Evolution of loss of heterozygosity patterns in hybrid genomes of Candida yeast pathogens
Source: BMC Biol. 2023 May 11;21:105. doi: 10.1186/s12915-023-01608-z (PMC10173528; doi:10.1186/s12915-023-01608-z)
Supplement: Supplementary file 9 — Additional file 9. Mitochondrial inheritance and LOH in mtDNA-interacting pentatricopeptide repeat proteins. [file 12915_2023_1608_MOESM9_ESM.docx]

**Supplementary file 9. Mitochondrial inheritance and LOH in mtDNA-interacting pentatricopeptide repeat proteins (PPR).**

Different hybrid clades in *C. orthopsilosis* have retained mitochondria from the different parental lineages, which provides us with a unique opportunity to assess whether mito-nuclear incompatibilities are driving LOH patterns. Earlier work determined that *C. orthopsilosis* clades 1 and 3 inherited the mitochondrial genome of parental A (corresponding to the reference genome), while clade 2 presented mitochondria from parental B, and clade 4 had a recombinant mitochondrion [[18]](https://paperpile.com/c/whwDJf/T7wA). We here confirmed these results, and determined the origin of the mitochondria of the strains of *C. metapsilosis* clades 1.2 and 2 as the same as *C. metapsilosis* clade 1.1.

In a scenario of strong mito-nuclear incompatibility, we would expect the direction of LOH (i.e. which sub-genome is retained) in mtDNA interacting proteins to follow the same direction of mitochondrial inheritance (i.e. same parental genome would be retained to minimize negative epistatic interactions). Earlier studies have linked nuclear-encoded mitochondrial proteins (e.g. PPR) to incompatibilities in hybrid yeast species [[27]](https://paperpile.com/c/whwDJf/w01Y). We analyzed in detail thirteen genes predicted to encode PPR proteins in *C. metapsilosis* and *C. orthopsilosis* (Supplementary file 8). Our results showed that, except for *C. metapsilosis* (including both hybridization events) and *C. orthopsilosis* clade 4, most PPR proteins underwent full LOH in the majority of the strains. However, in the same *C. orthopsilosis* clade different PPRs lost their heterozygosity towards different parentals, and even the same PPR underwent LOH towards different parentals in clades 1 and 3 (which retained the same mitochondrial background). These results indicate that the direction of LOH in PPR proteins does not follow the mitochondrial inheritance in *C. orthopsilosis* hybrids (Supplementary file 8). Considering that all these strains are well succeeded and some of them have been kept in the laboratory for years, these results can possibly indicate that homozygosity in these genes is important, but that the specific retained allele is not so relevant for hybrid survival. This is in agreement with what was previously suggested for the nuclear genome of these species that shows no preferential retention of any of the two parental sub-genomes [[12, 16, 18]](https://paperpile.com/c/whwDJf/f1gv+Ku8l+T7wA), and with a recent analysis of *Saccharomyces* hybrids that revealed that the overall direction of LOH is not correlated with the direction of mitochondrial inheritance [[39]](https://paperpile.com/c/whwDJf/6aJy).

We analyzed in more detail two PPR genes for which experiments in other yeasts have proven a role in hybrid incompatibilities. For instance, *AEPep3* was previously shown to be responsible for incompatibilities in *Saccharomyces cerevisiae* x *Saccharomyces pastorianus* hybrids [[27]](https://paperpile.com/c/whwDJf/w01Y). We found that this gene is 100% homozygous in all *C. orthopsilosis* strains, except s424 and MCO471, and in all *C. metapsilosis* strains of clade 1 but not in the strain of clade 2 (Supplementary file 8). This suggests a selection for LOH in this gene, but also its non-essentiality. Furthermore, *CCMcm1* which was previously suggested to be a source of mito-nuclear incompatibilities in *S. cerevisiae* x *Saccharomyces bayanus* hybrids [[27]](https://paperpile.com/c/whwDJf/w01Y) was found to be always within LOH blocks in all *C. metapsilosis* isolates of clade 1.1, and all *C. orthopsilosis* clade 2. As all clades of *C. orthopsilosis* were originated from the cross of the same two lineages, thus presenting at the time of hybridization similar nuclear content, only differing in the mitochondrial genome, we hypothesized that, contrarily to what was observed for *S. cerevisiae* x *S. bayanus* hybrids [[27]](https://paperpile.com/c/whwDJf/w01Y), in *C. orthopsilosis* hybrids, the incompatibility related to *CCMcm1* could be asymmetric, i.e. only occurs if parental B mitochondria is retained. As the same authors showed that the strength of the incompatibility was dependent on carbon source, an alternative scenario would be that *C. metapsilosis* clade 1.1 and *C. orthopsilosis* clade 2 were under similar stresses during part of their evolution, selecting LOH in similar genes, such as *CCMcm1*. This last scenario would justify why, despite having the same mitochondrial mitotype, this gene is not homozygous in *C. metapsilosis* clades 1.2 and 2.
