# Supplementary figures and images for: Evolution of loss of heterozygosity patterns in hybrid genomes of Candida yeast pathogens
Source: BMC Biol. 2023 May 11;21:105. doi: 10.1186/s12915-023-01608-z (PMC10173528; doi:10.1186/s12915-023-01608-z)

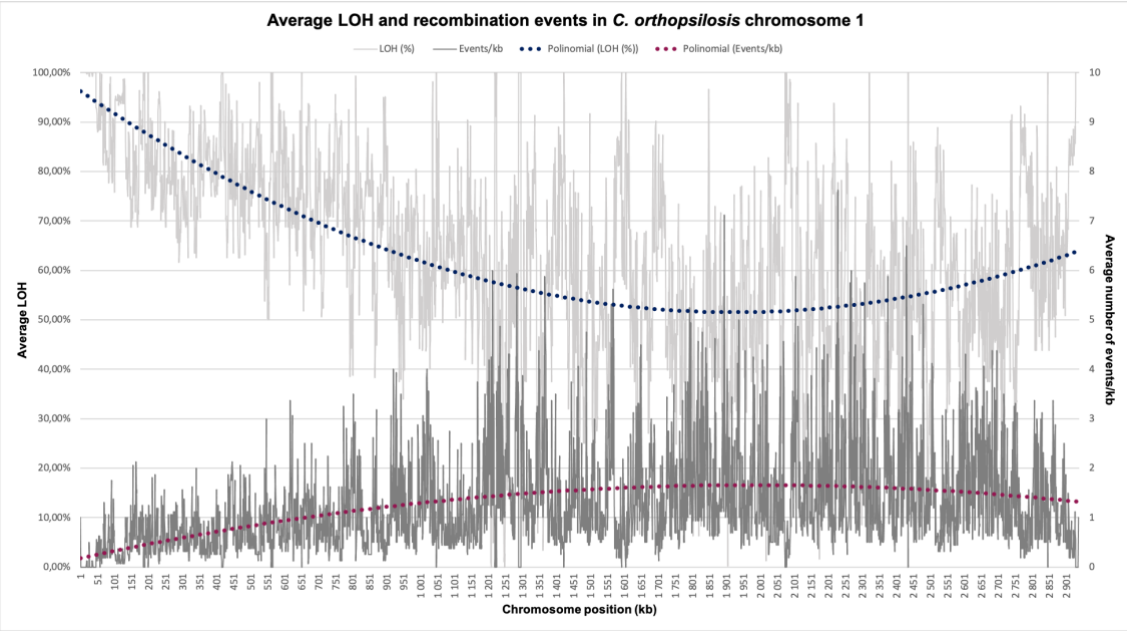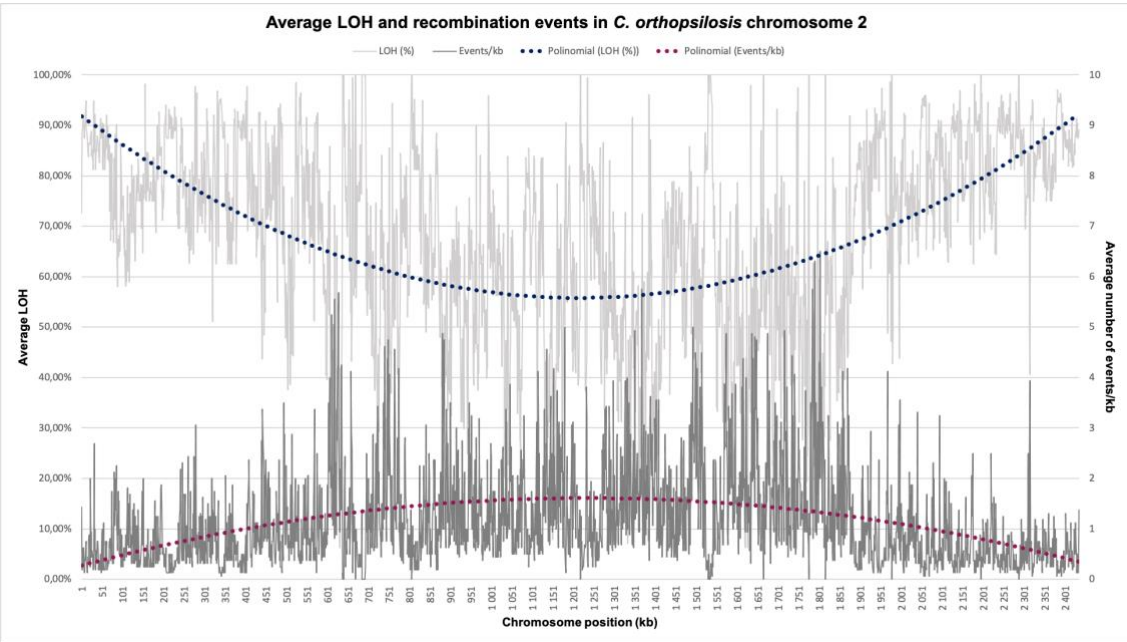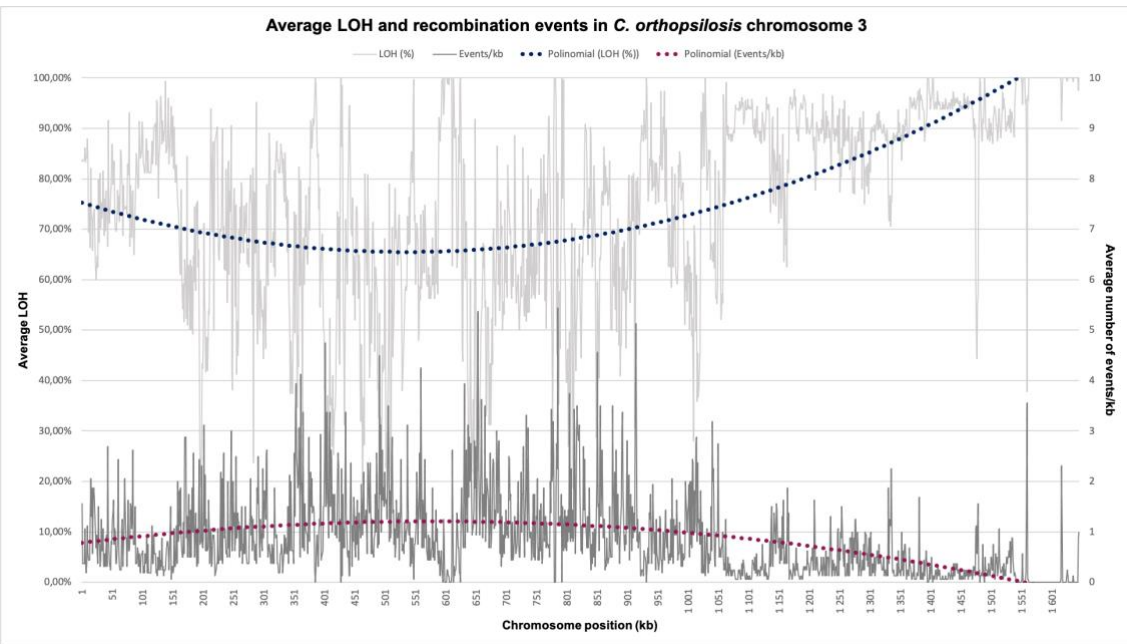

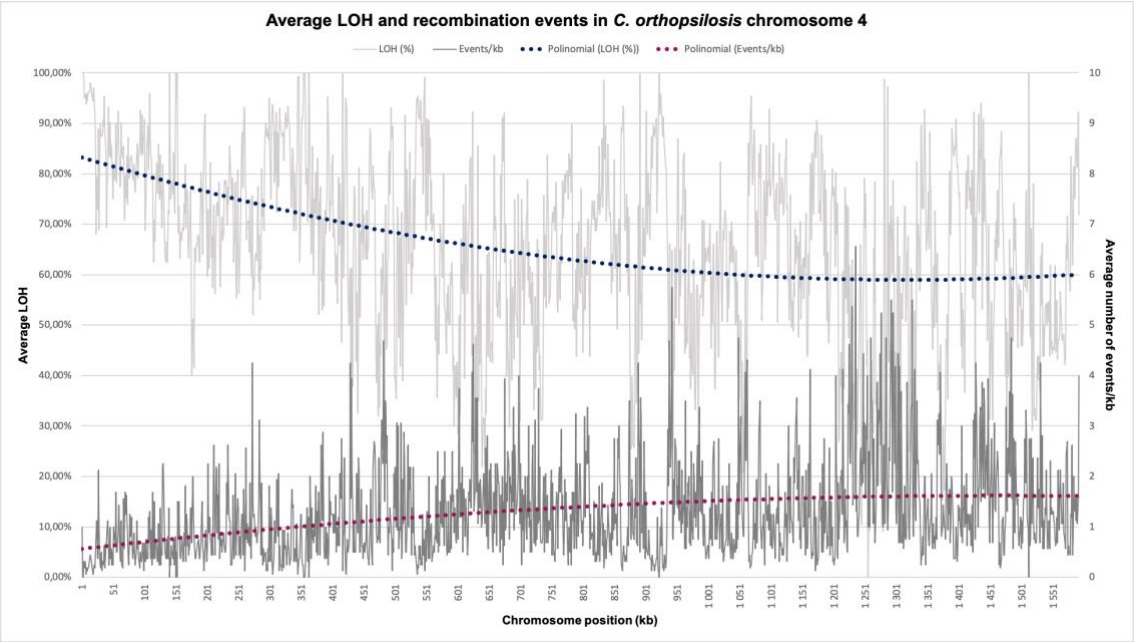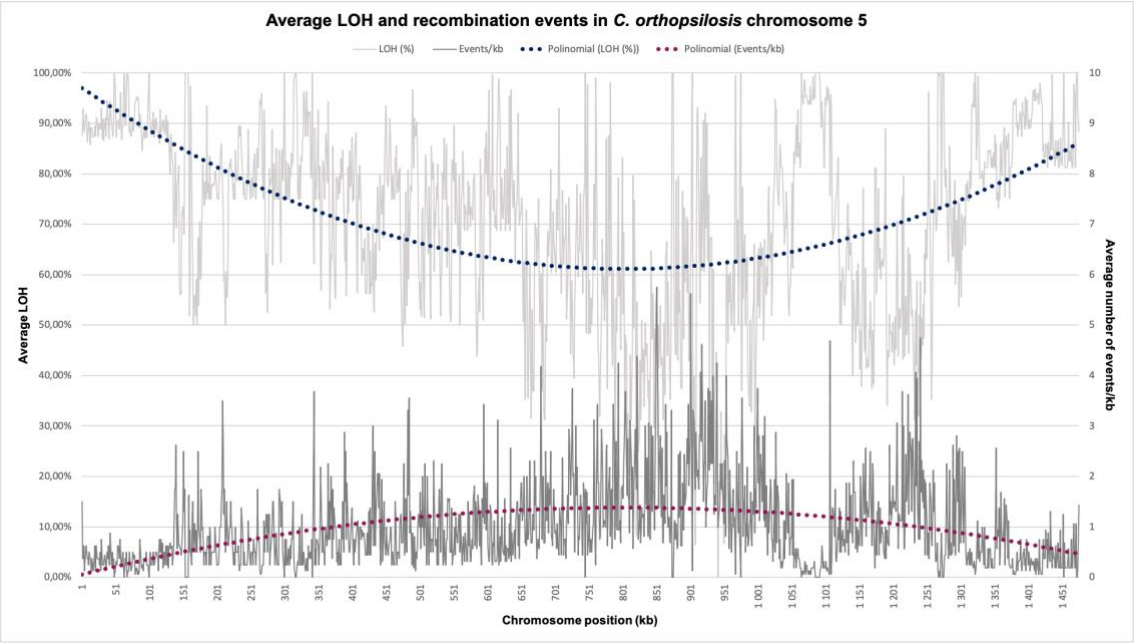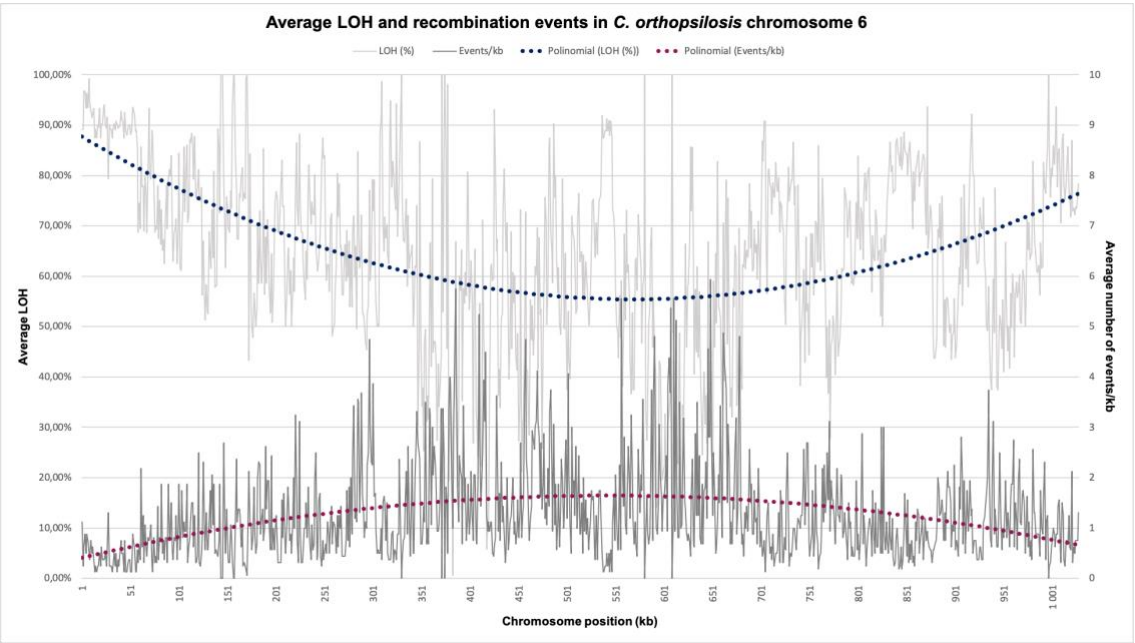

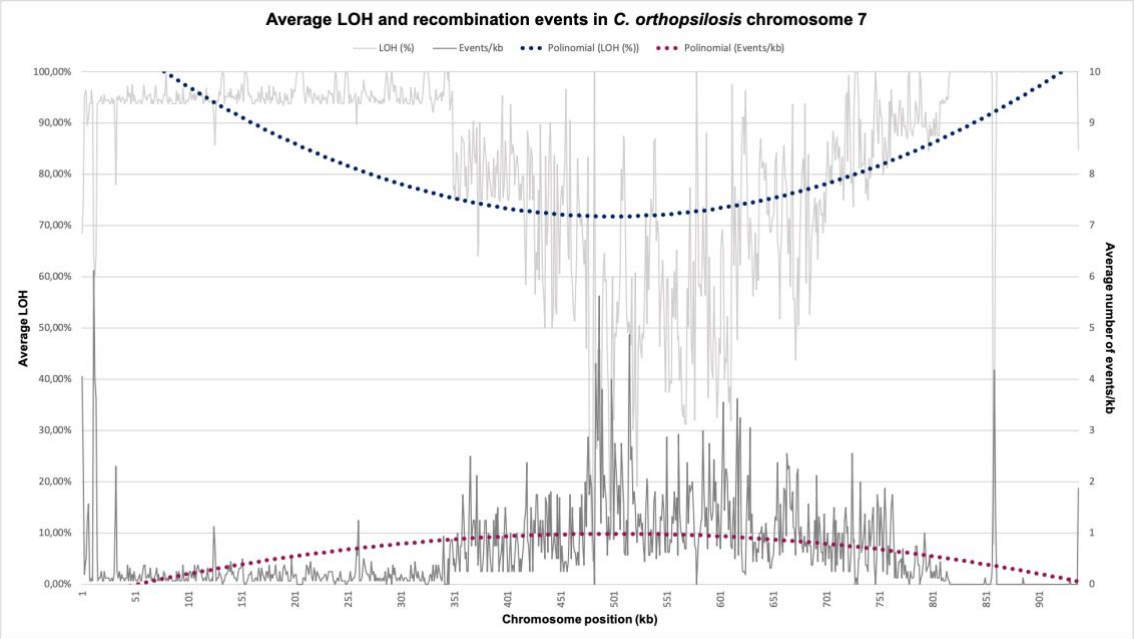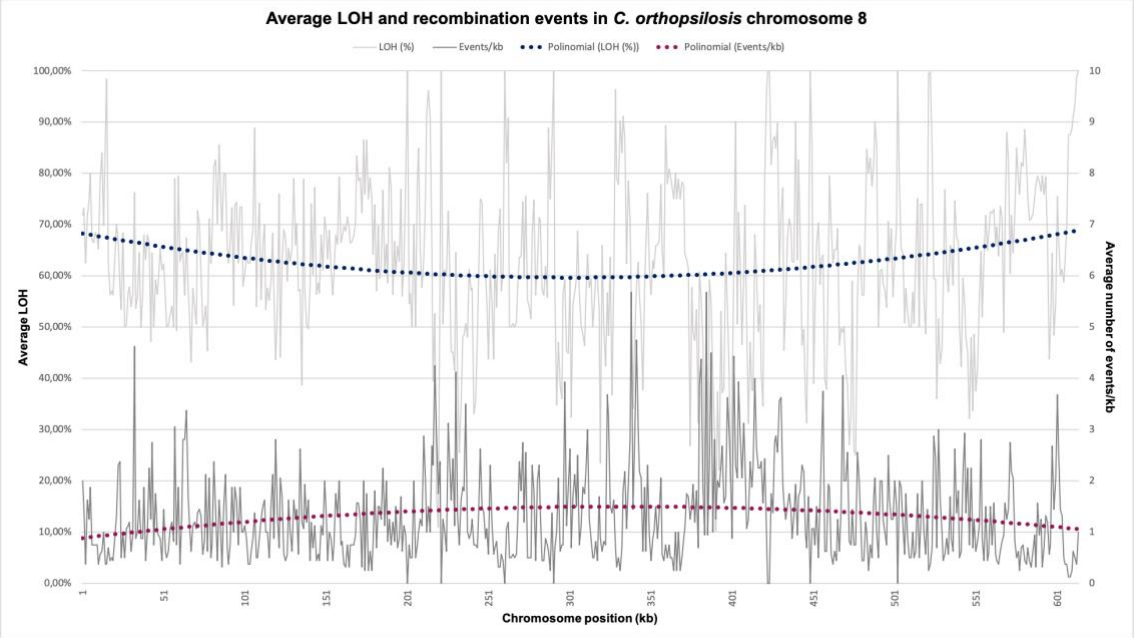

Supplement: Supplementary file 15 — Additional file 15. Average percentage of LOH and number of recombination events in 1 kb windows of all C. orthopsilosis chromosomes, only considering the 16 randomly selected strains represented in Fig. 2b to avoid bias of different clade sampling size. [file 12915_2023_1608_MOESM15_ESM.pdf]

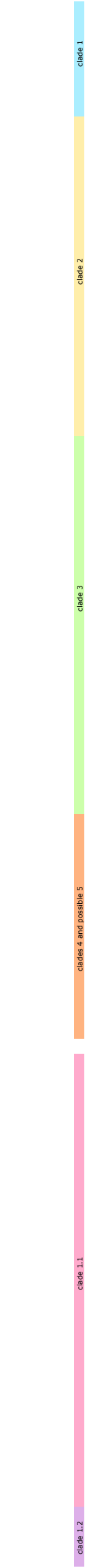

Supplement: Supplementary file 21 — Additional file 21: Fig. S1. Pairwise comparisons in C. orthopsilosis and C. metapsilosis strains. The different hybrid clades are highlighted with the same colors as in Fig. 3. [file 12915_2023_1608_MOESM21_ESM.pdf]
