# Supplementary material for: Evolution of loss of heterozygosity patterns in hybrid genomes of Candida yeast pathogens
Source: BMC Biol. 2023 May 11;21:105. doi: 10.1186/s12915-023-01608-z (PMC10173528; doi:10.1186/s12915-023-01608-z)

Supplementary figure 2. Phylogenetic tree of the reconstructed ITS and 26S sequence alignment of all *C. metapsilosis* strains and publicly available sequences of environmental isolates. Trees were rooted using *C. parapsilosis* as outgoup.

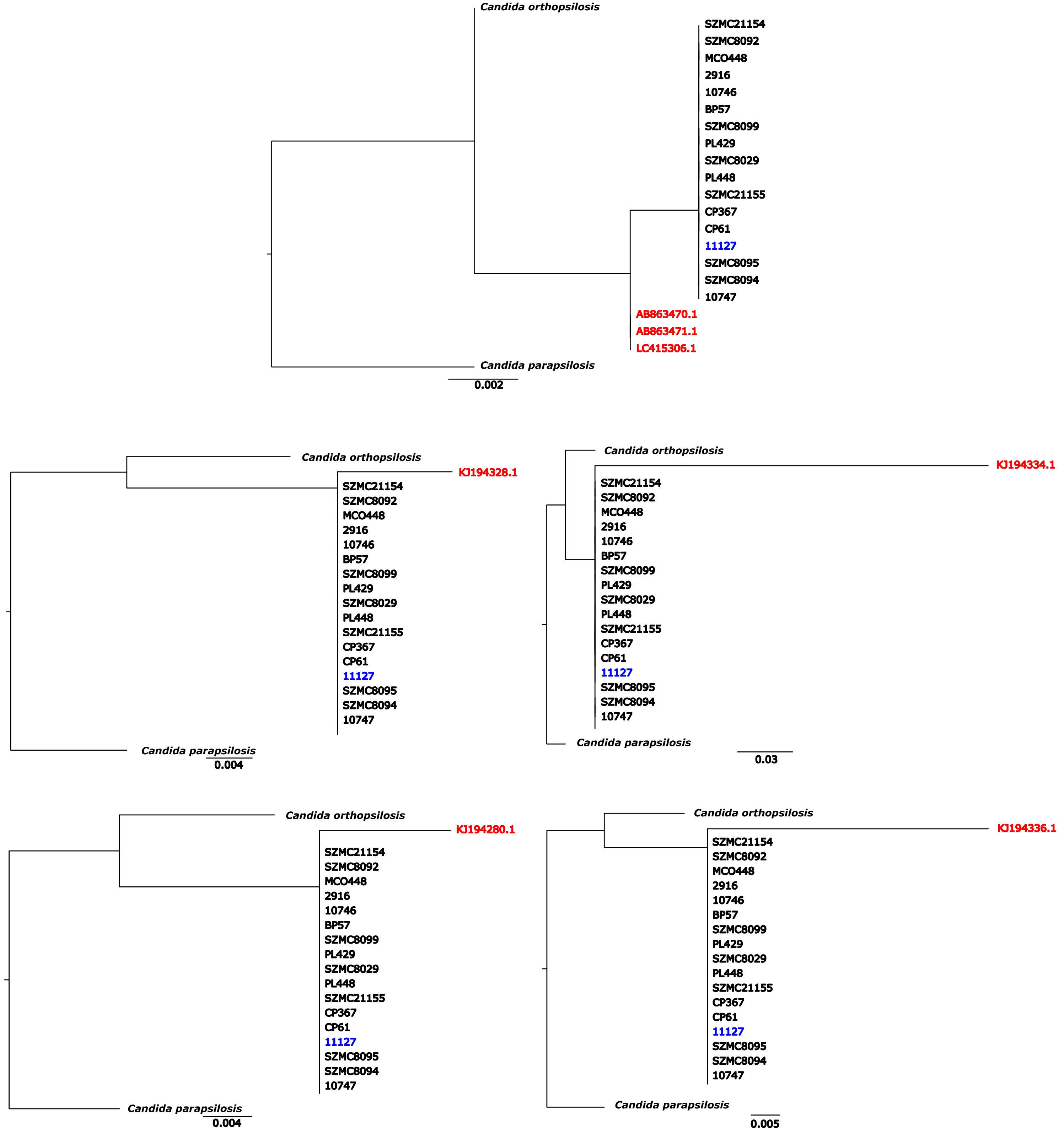

Supplement: Supplementary file 22 — Additional file 22: Fig. S2. Phylogenetic tree of the reconstructed ITS and 26S sequence alignment of all C. metapsilosis strains and publicly available sequences of environmental isolates. Sequences of environmental isolates retrieved from NCBI are highlighted in red, while the sequence of the environmental isolate sequenced in this study is highlighted in blue. Trees were rooted using C. parapsilosis as outgroup. [file 12915_2023_1608_MOESM22_ESM.pdf]
