# Supplementary material for: Evolution of loss of heterozygosity patterns in hybrid genomes of Candida yeast pathogens
Source: BMC Biol. 2023 May 11;21:105. doi: 10.1186/s12915-023-01608-z (PMC10173528; doi:10.1186/s12915-023-01608-z)

Supplementary file 3. IGV screenshots of the read alignment of strains CBS 2916 (clade 1.2), CBS 10747 (clade 1.1) and CP367 (clade 1.1) in *C. metapsilosis* genome assembly.

a) Different boundaries between the clades 1.1 and 1.2 in the recombination event in *C. metapsilosis MAT* locus.

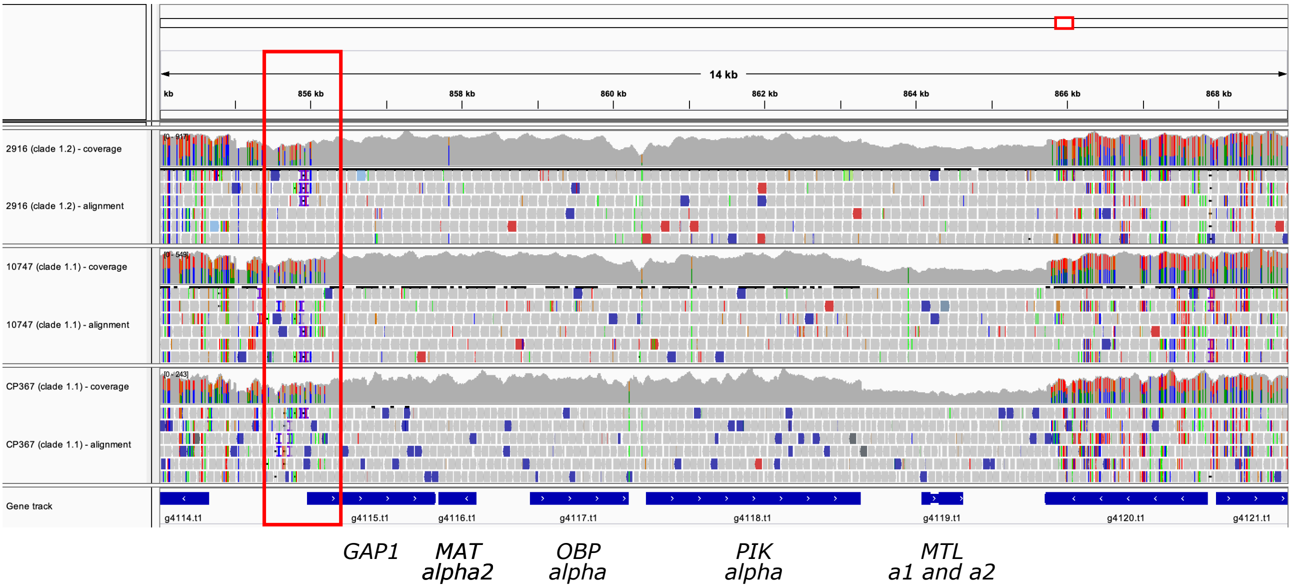

b) LOH blocks shared by clades 1.1 and 1.2 of *C. metapsilosis* covering the *MTC5* gene (g25)

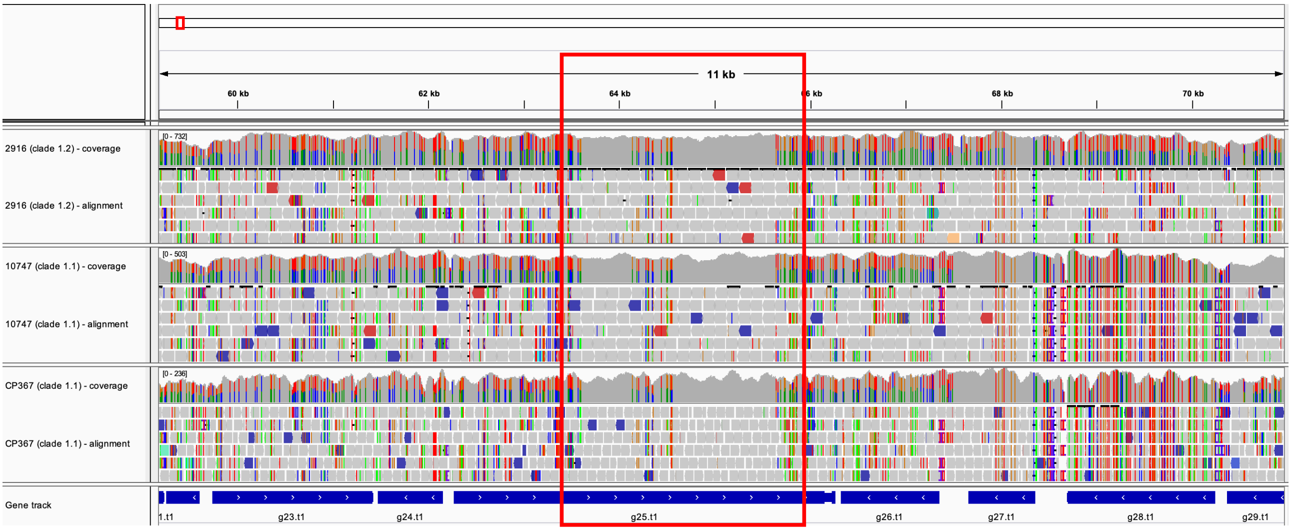

c) LOH blocks shared by clades 1.1 and 1.2 of *C. metapsilosis* covering the BPH1 gene (g211)

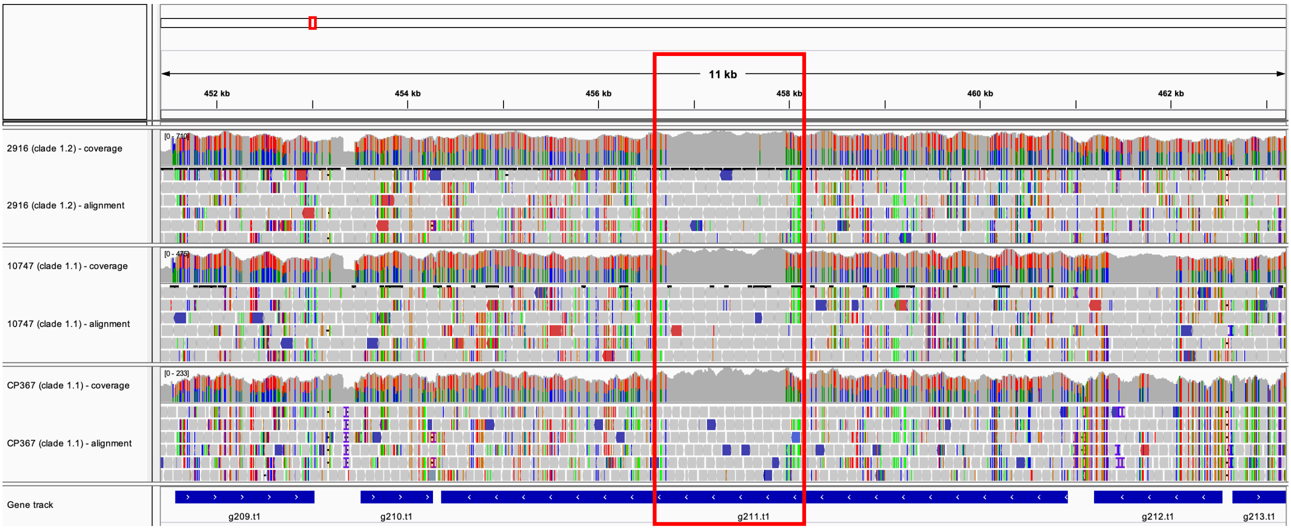

Supplement: Supplementary file 23 — Additional file 23: Fig. S3. IGV screenshots of the read alignment of strains CBS 2916, CBS 10747 and CP367 in C. metapsilosis genome assembly. a) MAT locus region of C. metapsilosis genome assembly, where an LOH block is flanked by heterozygous regions. The different boundaries in the left side of the LOH block are highlighted with a red box. b) LOH blocks shared by clades 1.1 and 1.2 of C. metapsilosis covering the MTC5 gene; c) LOH blocks shared by clades 1.1 and 1.2 of C. metapsilosis covering the BPH1 gene. [file 12915_2023_1608_MOESM23_ESM.pdf]
